# ADAMTS13 regulates angiogenic markers via Ephrin/Eph signaling in human mesenchymal stem cells under serum-deprivation stress

**DOI:** 10.1038/s41598-023-51079-z

**Published:** 2024-01-04

**Authors:** Srishti Dutta Gupta, Malancha Ta

**Affiliations:** Department of Biological Sciences, Indian Institute of Science Education and Research, Kolkata (IISER Kolkata), Mohanpur Campus, Dist: Nadia, Kolkata, West Bengal 741246 India

**Keywords:** Cell biology, Stem cells

## Abstract

Mesenchymal stem cells (MSCs) are known to facilitate angiogenesis and promote neo-vascularization via secretion of trophic factors. Here, we explored the molecular mechanism adopted by ADAMTS13 in modulating the expression of some key angiogenic markers in human umbilical cord-derived MSCs under serum-deprivation stress. Wharton’s jelly MSCs (WJ-MSCs) were isolated from the perivascular region of human umbilical cords by explant culture. ADAMTS13 was upregulated at both mRNA and protein levels in WJ-MSCs under serum-deprivation stress. Correspondingly, some key angiogenic markers were also seen to be upregulated. By screening signaling pathways, p38 and JNK pathways were identified as negative and positive regulators for expression of ADAMTS13, and the angiogenic markers, respectively. Our results also indicated the Notch pathway and p53 as other probable partners modulating the expression of ADAMTS13 and the angiogenic markers. Knockdown of ADAMTS13 using siRNA led to reversal in the expression of these angiogenic markers. Further, ADAMTS13 was shown to act via the EphrinB2/EphB4 axis followed by ERK signaling to control expression of the angiogenic markers. Interestingly, stronger expression levels were noted for ADAMTS13, VEGF and PDGF under a more stringent nutrient stress condition. Thus, we highlight a novel role of ADAMTS13 in WJ-MSCs under nutrient stress condition.

## Introduction

Mesenchymal stem cells (MSCs) are known to possess immunomodulatory capacity, secrete bioactive and trophic factors to promote angiogenesis and neo-vascularization and have innate migration potential to sites of injury, making them ideal candidates for treating many cardiovascular, neurodegenerative and immune-mediated diseases^1^.

Unfortunately, MSC-based therapeutic efficacy gets challenged due to the hostile micro-environment, marked by nutrient-deprivation, hypoxia or an inflammatory milieu, that the transplanted cells have to encounter at the injured site as demonstrated by many clinical and pre-clinical studies^2^. Blood supply disruption leading to nutrient limitation is one major challenge that MSCs face at diseased tissue or injury sites. All these could lead to reduced functionality of the MSCs and ultimately, impaired paracrine signaling and regenerative capacity.

Thus, it gets imperative to investigate the angiogenic potential of MSCs in response to nutrient-deprivation stress in order to maximize the clinical benefits of MSC-based therapies. Some important angiogenic factors that may be secreted by MSCs include vascular endothelial growth factor (VEGF), platelet derived growth factor (PDGF), hepatocyte growth factor (HGF), basic fibroblast growth factor (bFGF), angiopoietin-1 (ANGPT1), interleukin-6 (IL-6) etc.^3^.

ADAMTS13, which is primarily synthesized in hepatic stellate cells and in small amounts in endothelial cells, is a disintegrin and metalloproteinase with thrombospondin type I motif known for cleaving the von-Willebrand factor (vWF)^4^. Apart from its conventional role in thrombosis, ADAMTS13 has been shown to be associated with angiogenesis, inflammation and degradation of the extracellular matrix (ECM)^5,6^. Furthermore, ADAMTS13 is lately being studied for its roles beyond the proteolytic action on vWF^6–8^. A recent report demonstrated that ADAMTS13 was required for neo-vascularization and vascular repair in mice following ischemic stroke^9^. ADAMTS13 and certain variants of ADAMTS13 have been reported to promote angiogenesis through the VEGF-VEGFR2 signaling pathway^6^. However, a contradictory report suggested that exogenous recombinant human ADAMTS13 inhibited angiogenesis in a dose-dependent manner in VEGF-rich medium^10^.

MSCs can be harvested from a wide variety of tissue sources^11^, and depending on the tissue source, the functionality and the therapeutic potential of the MSCs may vary. Wharton’s jelly (WJ) of human umbilical cords, which are considered medical waste, represent a convenient source for cellular therapies as they involve non-invasive painless collection. Being a ‘young’ source, the MSCs isolated from them exhibit unique immunomodulatory properties and higher proliferation potential compared to some of the adult sources^12^. In MSCs, it has been reported previously that serum-deprivation led to an upregulation in the expression of genes associated with angiogenesis and endothelial differentiation^13^. Moreover, it has also been reported that an ischemia-like stress condition, comprising of glucose-deprivation in combination with a hypoxic micro-environment, led to elevated levels of expression of pro-angiogenic factors in adipose-derived MSCs^14^. Thus, determining the key factors and the underlying pathways involved in regulating the angiogenic potential of WJ-MSCs under conditions of nutrient-deprivation becomes imperative.

In this work, we focussed mainly on serum-deprivation stress and demonstrated a strong upregulation of ADAMTS13 expression in WJ-MSCs under serum-deprived condition. Correspondingly, it was seen that the expression of certain potent angiogenic markers, were upregulated. Further, the p38 and the JNK pathways were identified as negative and positive regulators of ADAMTS13 expression, respectively. Knockdown of ADAMTS13 led to a reversal in the expression of the angiogenic markers, thus, further implicating its pro-angiogenic role. Finally, ADAMTS13’s upstream role to EphrinB2/EphB4 and ERK signaling axes in regulating the expression of these angiogenic markers was also established under the same conditions.

## Results

### Effect of serum-deprivation stress on expression of ADAMTS13 and some notable angiogenic markers

Isolated WJ-MSCs were characterised following the guidelines laid down by the International Society for Cellular Therapy (ISCT)^15^ (Supplementary Fig. S1a–d).

**Figure 1:**
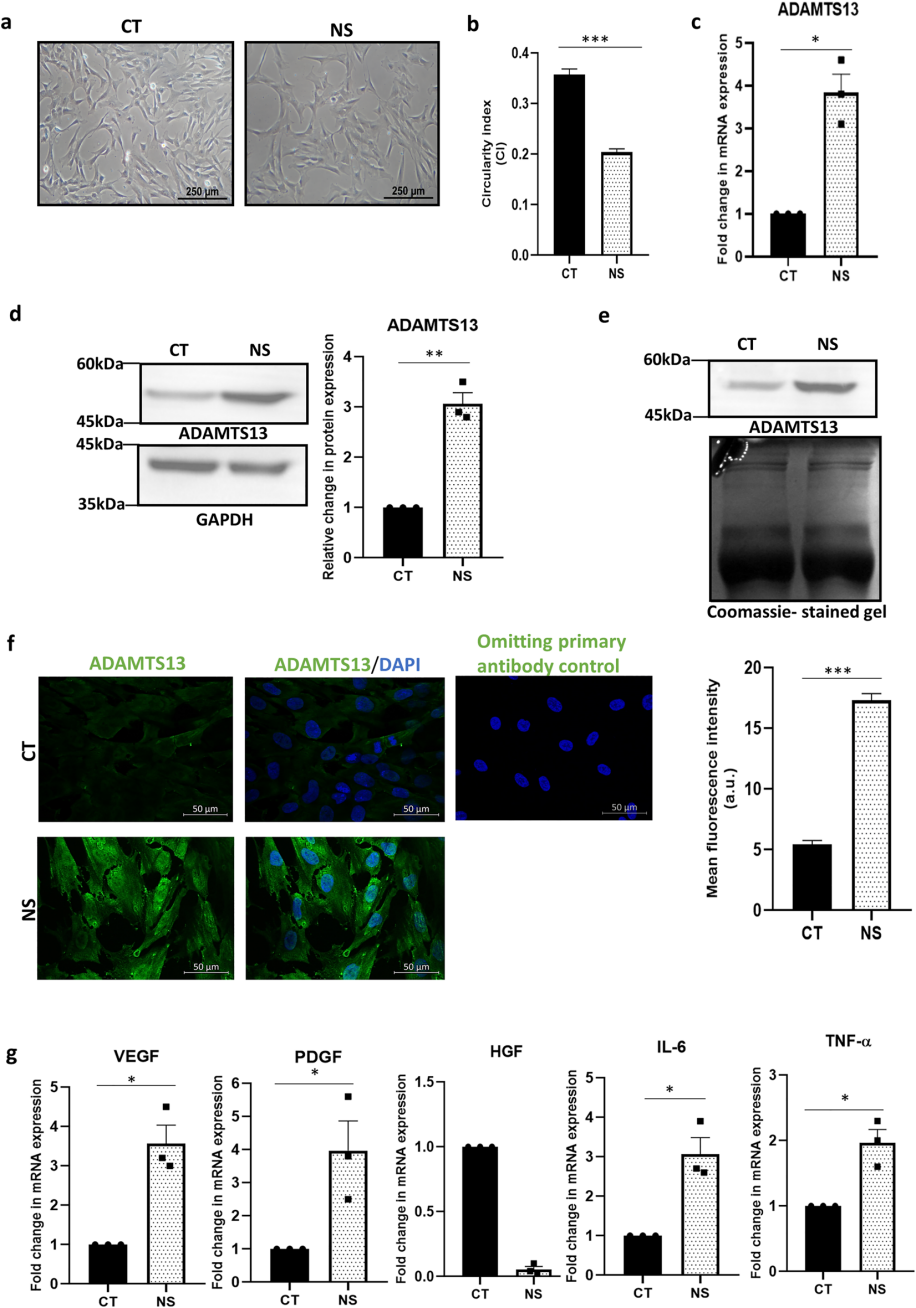
Effect of serum-deprivation on expression of ADAMTS13 and some notable angiogenic markers in WJ-MSCs. WJ-MSCs were subjected to serum-deprivation stress for 48 h. (**a**) Representative phase-contrast morphology images of control and serum-deprived WJ-MSCs under 10X magnification are displayed (n = 3). (**b**) Circularity index (CI) was quantified and compared for a total of 150 cells from 3 independent biological samples. (**c**) mRNA expression pattern of *ADAMTS13* was analysed by qRT-PCR for both control and serum-deprived WJ-MSCs (n = 3). *GAPDH* was used as an endogenous control to normalise the gene expression levels (**d**) Protein expression pattern of ADAMTS13 was detected by Western Blotting, representative image is shown (n = 3). Band intensity of protein expression levels of ADAMTS13 was quantified relative to GAPDH, which was used as a loading control and plotted. (**e**) Expression levels of ADAMTS13, secreted into the conditioned medium by WJ-MSCs, was detected by Western Blotting. SDS-PAGE separation of lyophilised conditioned medium followed by Coomassie brilliant blue staining was performed to display equal loading, representative image is shown (n = 3). (**f**) Representative immunofluorescence images depict distribution of ADAMTS13 (green) in control and serum-deprived WJ-MSCs. Nucleus was labelled with DAPI (blue). A sample without the primary antibody served as control. Mean fluorescence intensity for ADAMTS13 expression was quantified per cell using ImageJ software and plotted for a total of 70 cells (n = 70) for each experimental condition from two independent biological samples. (**g**) mRNA expression pattern of some key angiogenic markers (*VEGF, PDGF, HGF, IL-6* and *TNF-α*) was analysed and compared between control and serum-deprived WJ-MSCs by qRT-PCR (n = 3). *GAPDH* was used as the endogenous control to normalize the gene expression levels. Serum-deprived condition has been denoted as no-serum (NS) and control as CT in the figure. Each bar represents mean ± SEM (*p < 0.05, **p < 0.01, ***p < 0.001). Statistical comparisons were assessed using column statistics or two-tailed Student’s t-test. Original blots/gels are presented in Supplementary Fig. S7a,b.

On exposure to serum-deprivation stress, the WJ-MSCs assumed a thinner and elongated appearance as compared to WJ-MSCs under control condition, as reported earlier^16^ and confirmed once again here (Fig. 1a). This was affirmed by determining circularity index (CI), where the serum-deprived MSCs had a CI of 0.2 ± 0.006, while the CI of control WJ-MSCs measured to 0.35 ± 0.01 (*p* < 0.001) (Fig. 1b).

Next, the expression of ADAMTS13 was evaluated under serum-deprived condition and a strong upregulation in its expression was found both at the mRNA (*p* < 0.05) and protein levels (*p* < 0.01) (Fig. 1c,d). Increased ADAMTS13 level was noted even in the conditioned medium from serum-deprived WJ-MSCs, as compared to control (Fig. 1e). It is noteworthy that the ADAMTS13 protein detected in WJ-MSCs was of ~ 50 kDa size and not 150–190 kDa as typically reported in the literature^17^ (Supplementary Fig. S2).

To examine the localization pattern of ADAMTS13 in WJ-MSCs, immunofluorescence staining was performed. ADAMTS13 expression was noted majorly in the cytoplasm; and a relative increase in its expression was noted under serum-deprived condition (*p* < 0.001) (Fig. 1f). Correspondingly, the expression pattern of some key angiogenic markers was estimated and compared at the mRNA level under control and serum-deprived conditions (Fig. 1g). The angiogenic factors, *VEGF* and *PDGF* showed significant upregulation in their expression under serum-deprived condition (*p* < 0.05 for both). Certain inflammatory cytokines, known to have a pro-angiogenic role, like *IL-6* and *TNF-α* also showed upregulation in their expression under serum-deprived condition (*p* < 0.05 for both), while, interestingly, *HGF*, another potent angiogenic marker, showed a downregulation in expression.

### Identifying signaling pathways regulating the expression of ADAMTS13 and the angiogenic markers under serum-deprivation stress

Next, in order to analyse the signaling pathways which might be involved in regulating the expression of ADAMTS13 under serum-deprived condition in WJ-MSCs at the molecular level, specific small molecule inhibitors for blocking some relevant signaling pathways were used, and their impact on the expression of ADAMTS13 was assessed (Supplementary Fig. S3). It was noted that the p38 and JNK pathways, played considerable roles in regulating the expression of ADAMTS13 in WJ-MSCs.

**Figure 2:**
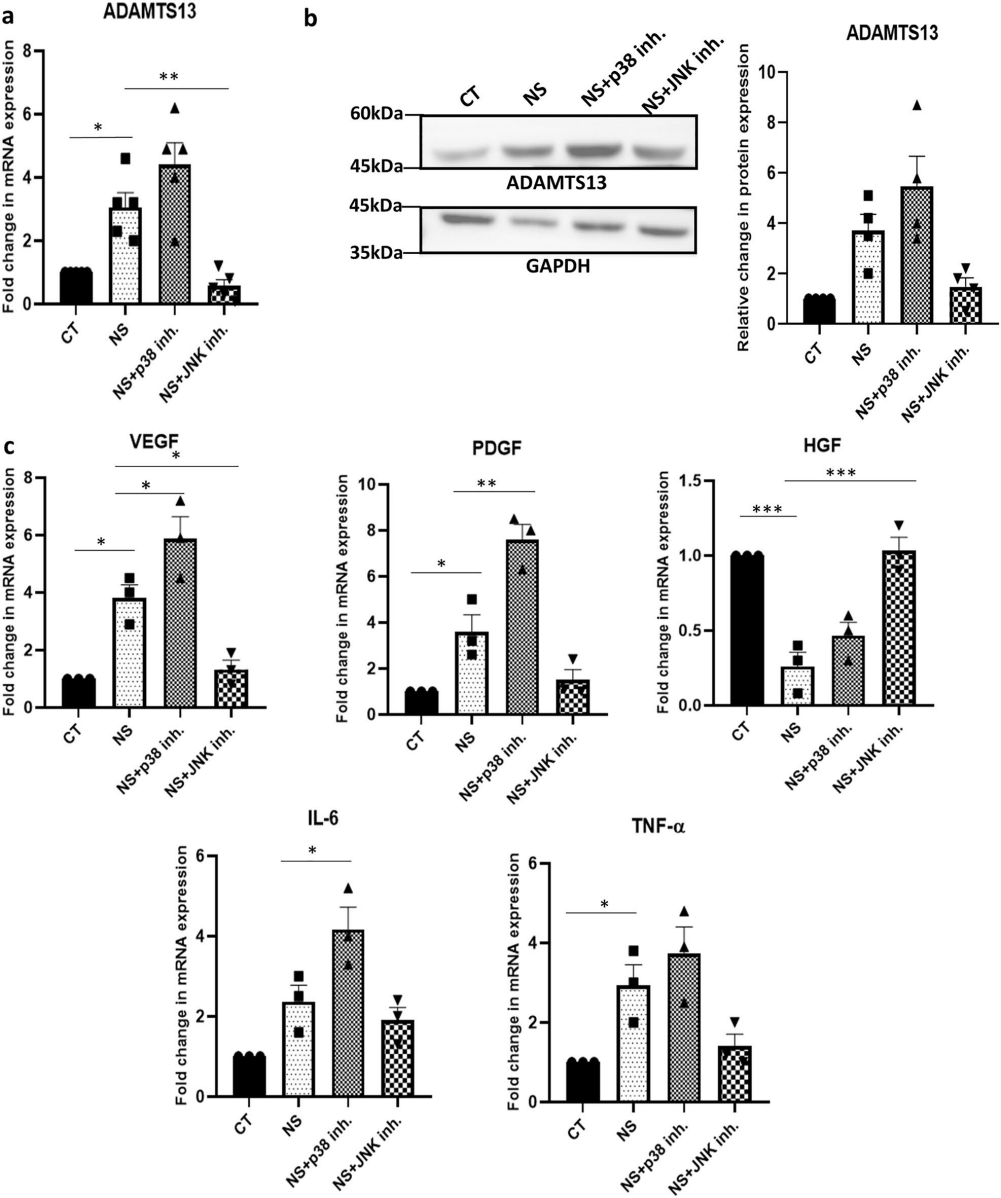
Identifying signaling pathways regulating ADAMTS13 expression and the notable angiogenic markers in serum-deprived WJ-MSCs. WJ-MSCs were cultured for 48 h under serum-deprived condition, in the presence of 10 μM of the p38 pathway inhibitor, SB203580 and 10 μM of the JNK pathway inhibitor, SP600125, individually. (**a**) mRNA expression pattern of *ADAMTS13* was analysed in the presence or absence of the inhibitors by qRT-PCR (n = 5). (**b**) Protein expression pattern of ADAMTS13 with p38 and JNK pathway inhibitions was detected by Western Blotting, representative image is shown (n = 4). Band intensity of protein expression levels of ADAMTS13 was quantified relative to GAPDH, which was used as a loading control and plotted. (**c**) mRNA expression pattern of the key angiogenic markers was analysed by qRT-PCR with p38 and JNK pathway inhibitions (n = 3). *GAPDH* was used as the endogenous control to normalize the gene expression levels in all the qRT-PCR studies. Serum-deprived condition has been denoted as no-serum (NS) and control as CT in the figure. Each bar represents mean ± SEM (*p < 0.05, **p < 0.01, ***p < 0.001). Statistical comparisons were assessed using one-way ANOVA followed by Bonferroni’s multiple comparison test. Original blots are presented in Supplementary Fig. S7c.

With inhibition of the p38 pathway, there was a further upregulation in the expression of *ADAMTS13* as compared to untreated WJ-MSCs; while with the inhibition of the JNK pathway, there was a downregulation in the expression of *ADAMTS13* (*p* < 0.01) (Fig. 2a). For further validation, the expression profile of ADAMTS13 was evaluated at the protein level, wherein with the inhibition of the p38 pathway, a further upregulation in the expression of ADAMTS13 was noted, as compared to untreated WJ-MSCs under serum-deprivation. Correspondingly, there was a downregulation in ADAMTS13 protein level with inhibition of the JNK pathway (Fig. 2b).

Next, the mRNA expression profile of some key angiogenic markers was assessed under serum-deprived condition with p38 or JNK pathway inhibitor treatment, individually. The angiogenic factors, *VEGF, PDGF, IL-6* and *TNF-α* showed a further upregulation in their mRNA expression with p38 pathway inhibition, while a downregulation in their expression was observed with JNK pathway inhibition as compared to untreated WJ-MSCs under serum-deprived condition. Interestingly, *HGF* again showed an opposite trend (Fig. 2c). Thus, these key angiogenic markers demonstrated the same pattern of regulation under serum-deprived condition as for ADAMTS13.

### Notch pathway and p53 as other regulators of ADAMTS13 and the angiogenic markers’ expression

**Figure 3:**
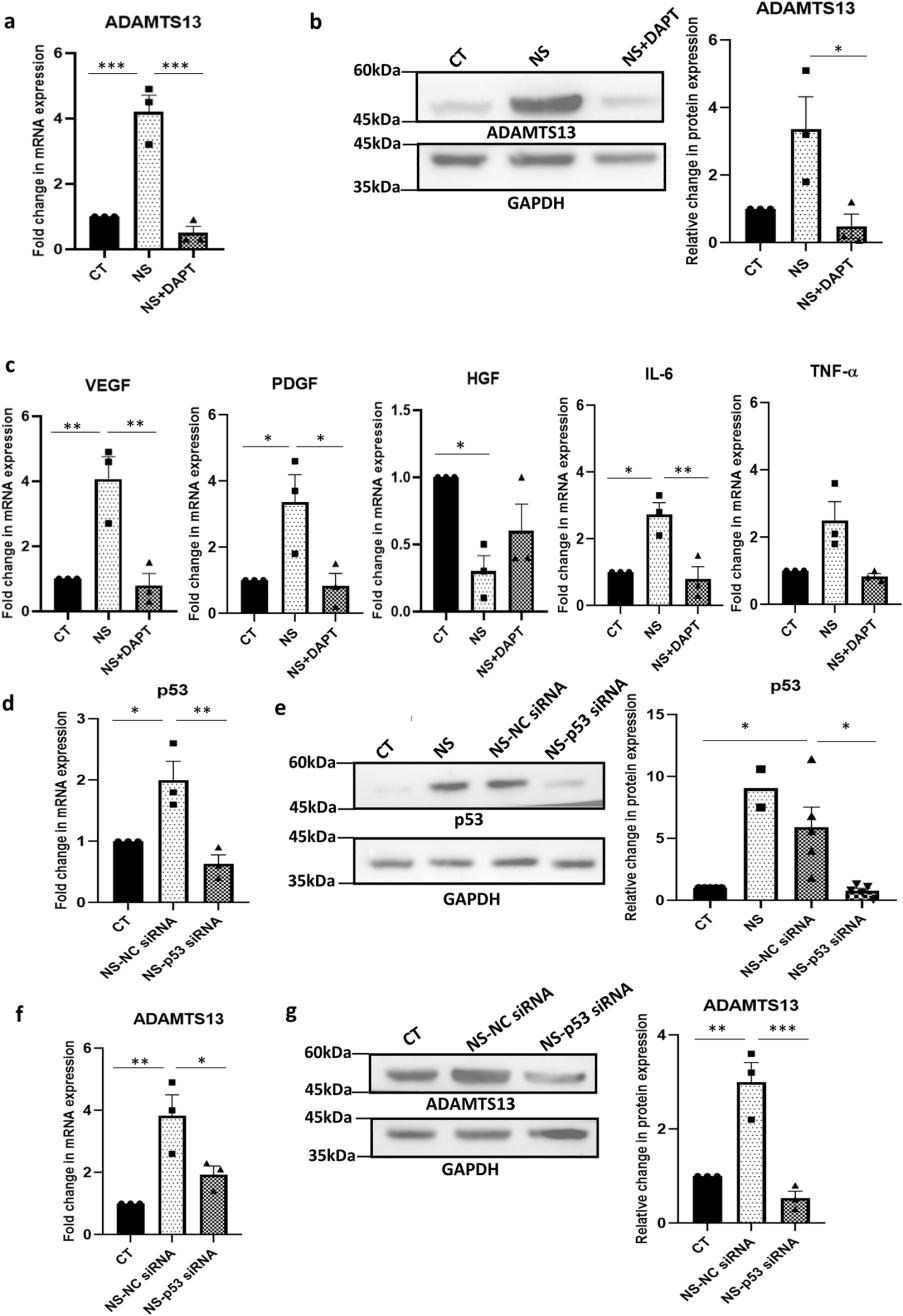

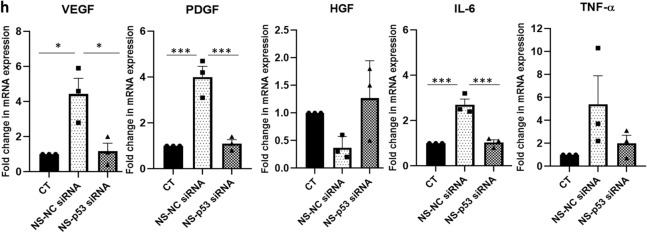
Notch pathway and p53 as other regulators of ADAMTS13 expression. WJ-MSCs were cultured for 48 h under serum-deprived condition, in the presence of 20 µM of Notch pathway inhibitor, DAPT, GSI-IX, LY-374973 (**a**) mRNA expression pattern of *ADAMTS13* with Notch pathway inhibition was analysed by qRT-PCR (n = 3). (**b**) Protein expression pattern of ADAMTS13 with Notch pathway inhibition was detected by Western Blotting, representative image is shown (n = 3). Band intensity of protein expression levels of ADAMTS13 was quantified relative to GAPDH, which was used as a loading control and plotted. (**c**) mRNA expression pattern of the key angiogenic markers, with Notch pathway inhibition was analysed by qRT-PCRs (n = 3). Impact of p53 knockdown on the expression of ADAMTS13 and the angiogenic markers. (**d**) The knockdown of *p53* following transfection was validated by qRT-PCR. *GAPDH* was used as the endogenous control (n = 3). (**e**) For further validation, protein-level expression of p53 following transfection was also analysed by Western Blotting and a representative image is shown (n = 3). Band intensity of protein expression levels of p53 was quantified relative to GAPDH, which was used as a loading control, and plotted. (**f**) mRNA and (**g**) protein expression levels of ADAMTS13 with p53 knockdown was evaluated by qRT-PCR and Western Blotting, respectively (n = 3). Representative blot image is shown. (**h**) mRNA expression pattern of the key angiogenic markers with *p53* knockdown was analysed by qRT-PCR (n = 3). *GAPDH* was used as the endogenous control to normalize the gene expression levels in all the qRT-PCR studies. Serum-deprived condition has been denoted as no-serum (NS), negative control as NC and control as CT in the figure. Each bar represents mean ± SEM (*p < 0.05, **p < 0.01, ***p < 0.001). Statistical comparisons were assessed using one-way ANOVA followed by Bonferroni’s multiple comparison test. Original blots are presented in Supplementary Fig. S7d–f.

Though Notch signaling is primarily known to play a vital role in the determination of cell fate, reports have also suggested its roles in promoting angiogenesis and vasculogenesis^18^. Hence, the effect of Notch pathway inhibition on the expression of ADAMTS13 and some key angiogenic markers in WJ-MSCs under serum-deprivation condition was analysed next. WJ-MSCs were treated with the Notch pathway inhibitor, N-[N-(3,5-difluorophenacetyl)-l-alanyl]-s-phenylglycinet-butyl ester (DAPT), under serum-deprived condition. On evaluating the mRNA expression profile of *ADAMTS13,* a significant downregulation was noted with DAPT treatment under serum-deprived condition (*p* < 0.001) (Fig. 3a). This was further validated at the protein level, where again a significant downregulation in the expression of ADAMTS13 was observed (*p* < 0.05) (Fig. 3b). Further, the expression profile of the angiogenic markers was assessed, where *VEGF, PDGF, IL-6* (all significant) and *TNF-α* also showed a downregulation in their mRNA expression in serum-deprived WJ-MSCs in the presence of DAPT (Fig. 3c); whereas *HGF* showed a small reversal in its expression. These results again corresponded with the ADAMTS13 profile.

Tumor suppressor gene, *TP53*, is known to play an important role in regulating various functions of cells, in response to a variety of stresses^19^. A report suggested that the p53 protein inhibited angiogenesis by interfering with the production of pro-angiogenic molecules and also increased the endogenous release of angiogenic inhibitors^20^. Hence, we also investigated whether p53 was regulating the expression of ADAMTS13 and the angiogenic markers in serum-deprived WJ-MSCs. For this siRNA mediated knockdown of *p53* in WJ-MSCs under serum-deprived condition was performed. The validation of p53 knockdown was carried out both at the mRNA and protein levels (Fig. 3d,e). The expression of ADAMTS13 was evaluated both at mRNA and protein levels in p53 knocked down WJ-MSCs under serum-deprivation. A significant downregulation in its expression was noted (Fig. 3f,g), hinting at the fact that p53 could be controlling the expression of ADAMTS13 in WJ-MSCs under serum-deprivation stress. Next, the expression profile of the same angiogenic markers was studied in p53 knocked down WJ-MSCs under serum-deprived condition. Corresponding to ADAMTS13 downregulation, *VEGF*, *PDGF, IL-6* (all significant) and *TNF-α*, showed a downregulation in their mRNA expression under serum-deprived condition with p53 knockdown; whereas *HGF*, exhibited an increase (Fig. 3h). These results indicated that p53 also could be modulating the expression of ADAMTS13, and in parallel affected the expression of the stated angiogenic markers in WJ-MSCs under serum-deprivation stress.

### Effect of ADAMTS13 knockdown on biological properties of MSCs and the expression of some notable angiogenic markers under serum-deprivation

**Figure 4:**
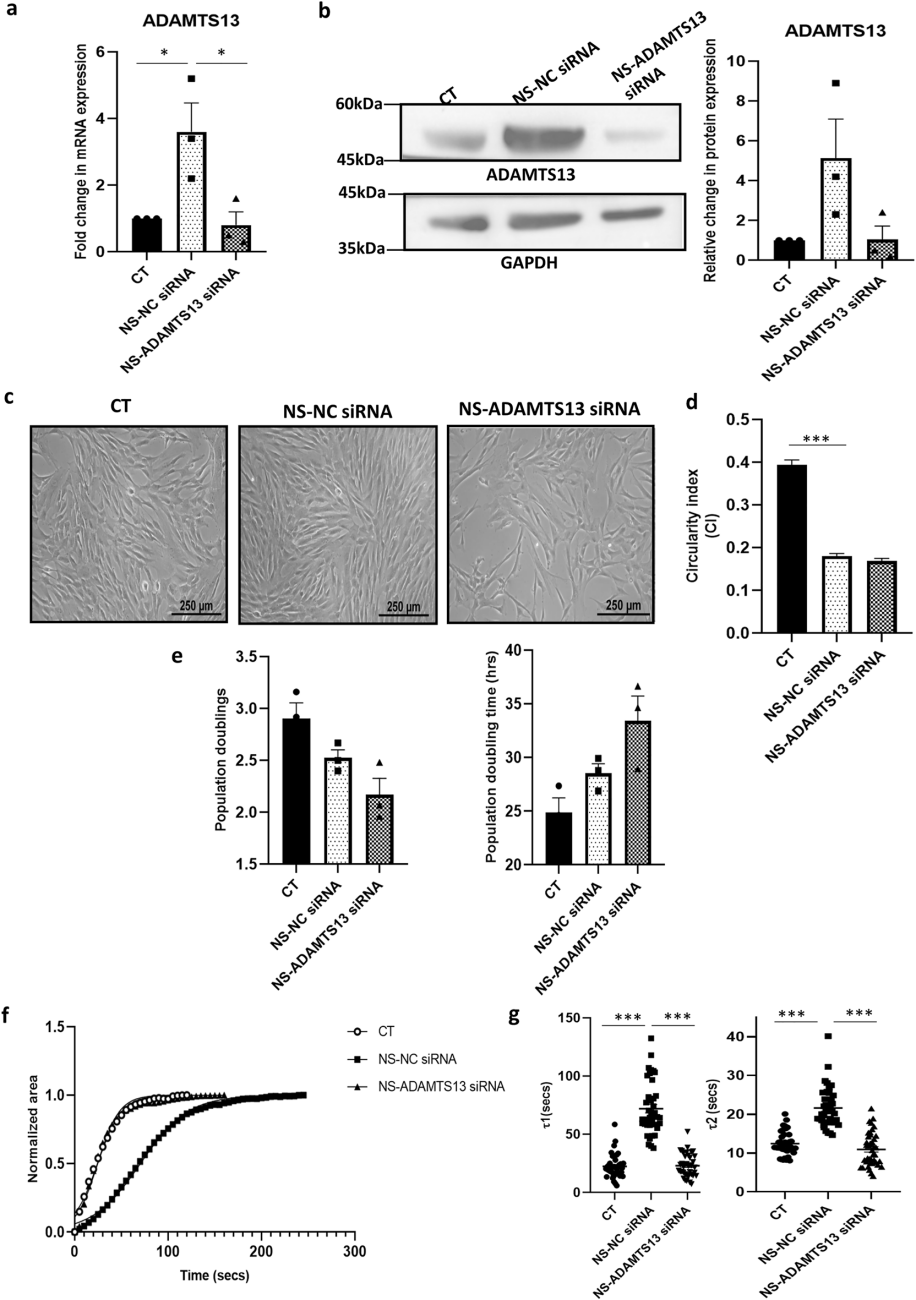

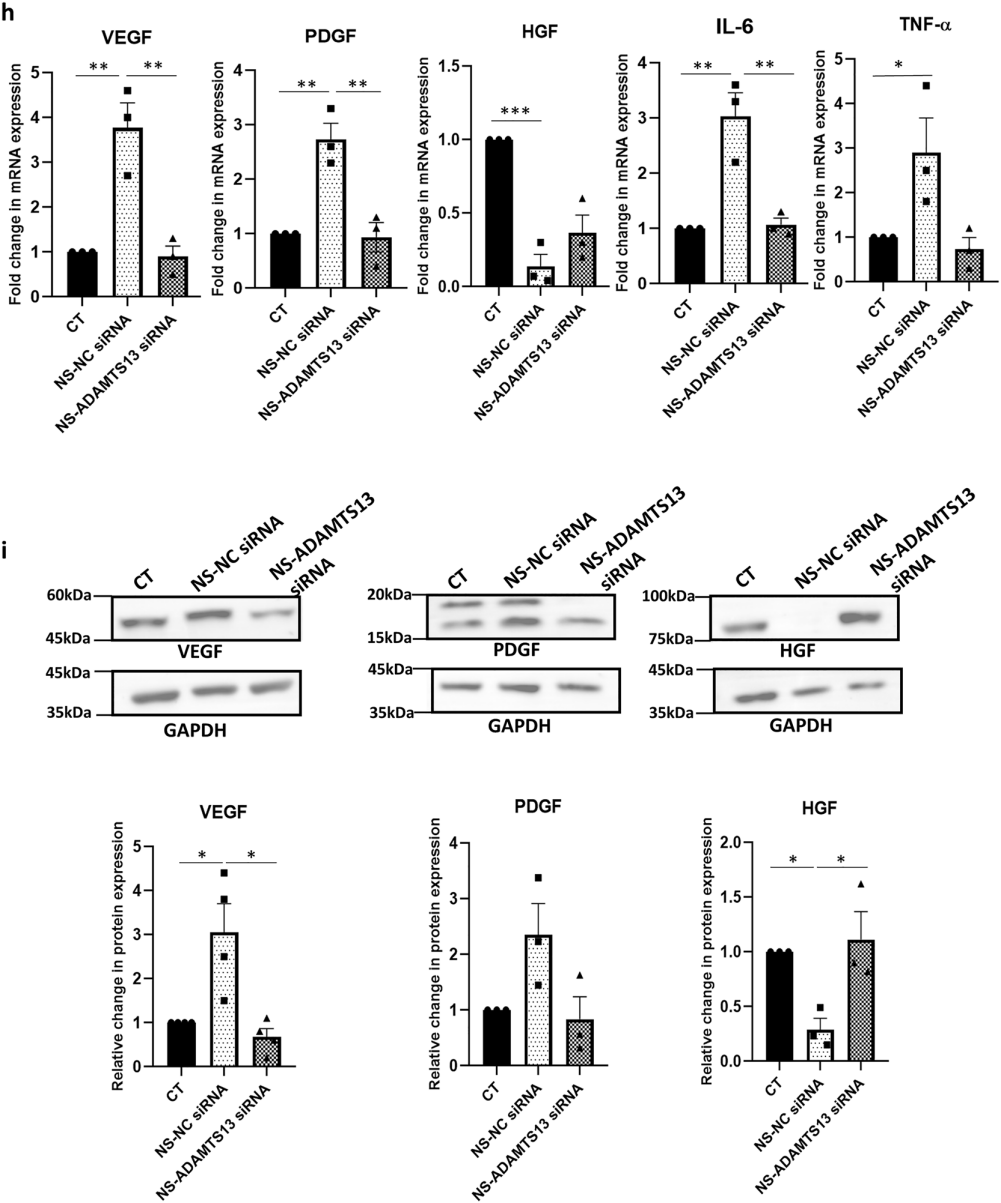
Effect of ADAMTS13 knockdown on basic characteristics and angiogenic marker expression of WJ-MSCs. WJ-MSCs transfected with *ADAMTS13* or NC siRNA were exposed to serum-deprivation stress for 48 h. (**a**) The knockdown of *ADAMTS13* was validated by qRT-PCR. *GAPDH* was used as the endogenous control (n = 3). (**b**) Protein level validation of the same was analyzed by Western Blotting, representative blot image being shown (n = 3). Band intensity of ADAMTS13 was quantified relative to GAPDH, which was used as a loading control. (**c**) Representative phase-contrast morphology images of WJ-MSCs transfected with *ADAMTS13* or NC siRNA under serum-deprivation stress for 48 h are displayed (n = 3). (**d**) Circularity index (CI) was compared between control WJ-MSCs and WJ-MSCs transfected with *ADAMTS13* or NC siRNA under serum-deprivation. A total of 100 cells per experimental condition from two independent biological samples, were quantified and compared. (**e**) For assessing influence of *ADAMTS13,* if any*,* on growth kinetics, the number of population doublings and population doubling time of WJ-MSCs transfected with *ADAMTS13* siRNA or NC siRNA under serum-deprivation condition were evaluated against control WJ-MSCs. (**f**) Impact of ADAMTS13 knockdown on de-adhesion dynamics of serum-deprived WJ-MSCs was analyzed. The normalized change in cell spread area vs time was fitted to Boltzmann sigmoidal equation and (**g**) the time constants τ1 and τ2, thus obtained, were plotted. A total of 40 cells per experimental condition from two independent biological samples were used. (**h**) mRNA expression pattern of the key angiogenic markers following ADAMTS13 knockdown was analysed by qRT-PCR (n = 3). *GAPDH* was used as the endogenous control. (**i**) The expression of angiogenic markers, VEGF, PDGF and HGF was also assessed at the protein level by Western blotting (n ≥ 3), under the same conditions and representative blot images are shown. Band intensities were quantified relative to GAPDH, used as a loading control, and plotted. Serum-deprived condition has been denoted as NS (no-serum), negative control as NC and control as CT in the figures. Each bar represents mean ± SEM (*p < 0.05, **p < 0.01, ***p < 0.001). Statistical comparisons were assessed using one-way ANOVA followed by Bonferroni’s multiple comparison test. Original blots are presented in Supplementary Fig. S7g,h.

To evaluate any specific role of ADAMTS13 in impacting certain cellular characteristics of WJ-MSCs, and in regulating the expression of angiogenic markers, siRNA mediated knockdown of *ADAMTS13* was performed in WJ-MSCs under serum-deprived condition. WJ-MSCs were transfected with *ADAMTS13* siRNA and exposed to serum-deprivation for 48 h. The validation of ADAMTS13 knockdown was performed both at the mRNA (*p* < 0.05) and protein levels (Fig. 4a,b). The ADAMTS13 knocked down WJ-MSCs had morphology similar to the serum-deprived cells transfected with NC siRNA (Fig. 4c). CI data confirmed the same (Fig. 4d).

On assessing growth kinetics, the ADAMTS13 knocked down WJ-MSCs were found to exhibit lesser number of mean population doublings (2.17 ± 0.15 vs 2.52 ± 0.07) with longer population doubling time (33.5 ± 2.3 vs 28.6 ± 0.8 h) compared to NC siRNA transfected WJ-MSCs under serum-deprivation, though the differences were not significant (Fig. 4e). In the meantime, control WJ-MSCs had mean population doublings of 2.9 ± 0.15 with population doubling time of 24.9 ± 1.3 h (Fig. 4e).

As ADAMTS proteases can remodel ECM, we next wanted to assess the impact of ADAMTS13 knock down on deadhesion dynamics of the cells. Interestingly, the delayed cell–matrix deadhesion noted in serum-deprived WJ-MSCs treated with NC siRNA underwent a distinct reversal on knocking down *ADAMTS13* with siRNA (Fig. 4f). Knockdown of ADAMTS13 resulted in a significant decrease in τ1 and τ2 values from 72.05 ± 3.61 to 23.07 ± 1.56 and 21.61 ± 0.81 to10.94 ± 0.69 respectively, as compared to NC siRNA transfected serum-deprived WJ-MSCs (Fig. 4g). This was obtained from the sigmoidal curve of de-adhesion dynamics data indicating a possible role of ADAMTS13 in promoting cellular adhesion under serum-deprived condition (Fig. 4f,g).

Next, the expression profile of the same angiogenic markers was investigated in ADAMTS13 knocked down WJ-MSCs under serum-deprived condition. *VEGF*, *PDGF, IL-6* (all significant) and *TNF-α* showed a downregulation in their mRNA expression in ADAMTS13 knocked down WJ-MSCs; *HGF* showed a reversal in its expression. (Fig. 4h). Validation of the same was performed at the protein level for VEGF (*p* < 0.05), PDGF and HGF (*p* < 0.05), which yielded the same results (Fig. 4i). These results further indicated that ADAMTS13 played a role in modulating the expression of certain potent angiogenic markers in WJ-MSCs under serum-deprivation stress.

### Impact of inhibiting EphrinB2/EphB4 signaling on angiogenic markers under serum-deprivation condition

The EphrinB2/EphB4 receptor tyrosine kinase axis has been shown to play a vital role in aiding the regeneration and maturation of blood vessels via its interplay with VEGF, thereby promoting angiogenesis^21,22^. For the same reason, EphrinB2 and EphB4 were identified as therapeutic targets for angiogenesis related diseases.

**Figure 5:**
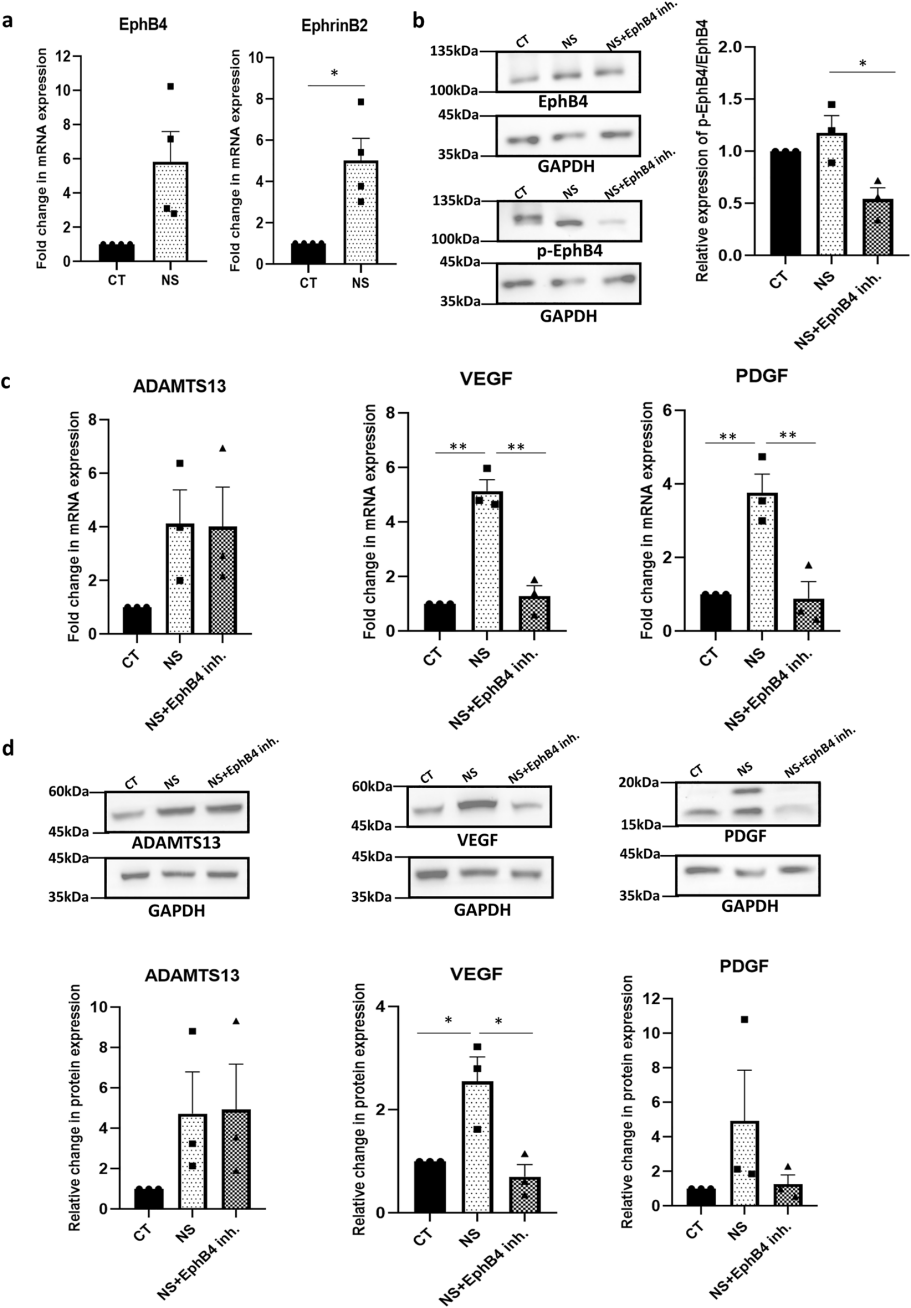

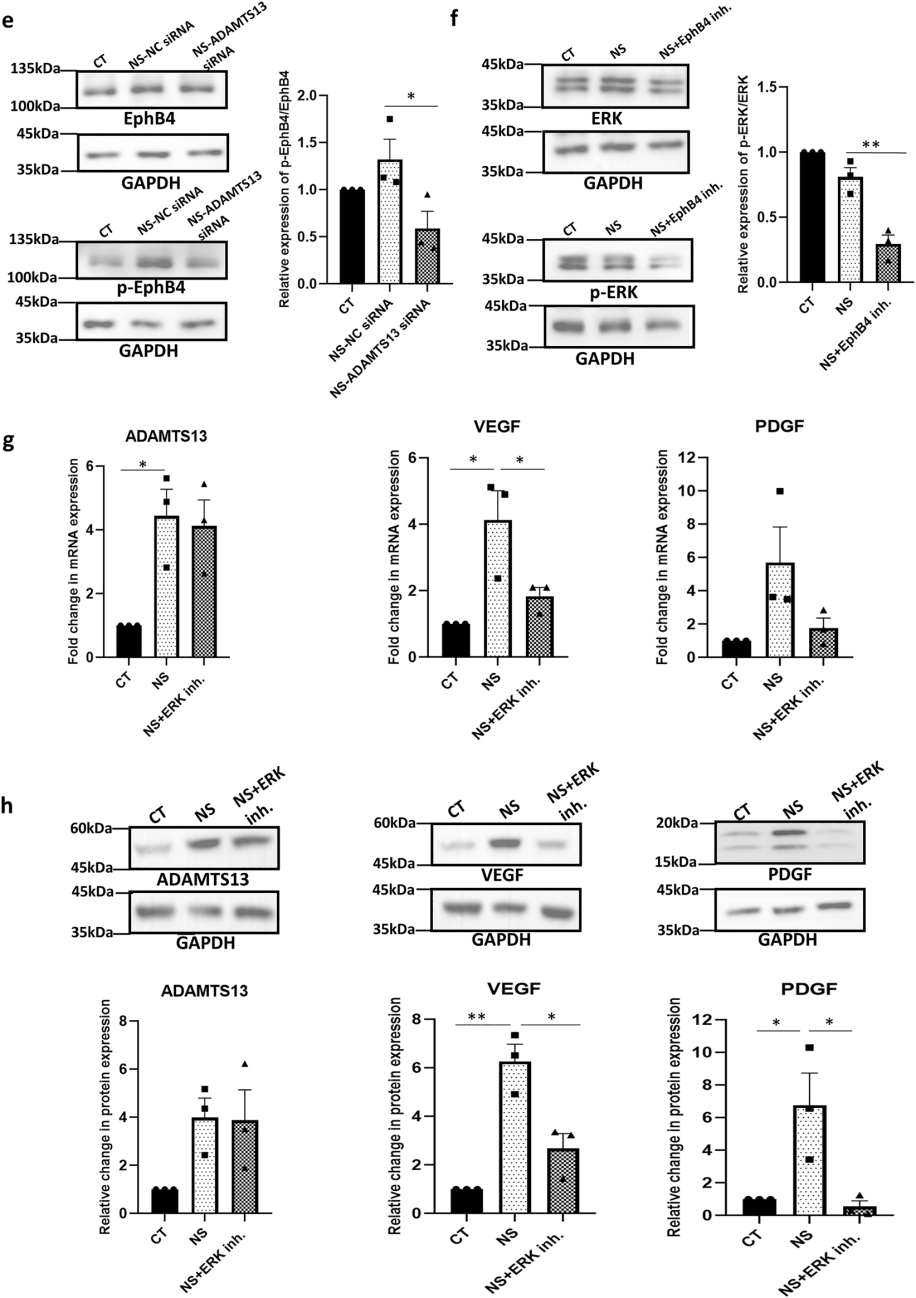
Influence of EphrinB2/EphB4 signaling on angiogenic markers in WJ-MSCs under serum-deprivation. (**a**) The mRNA expression levels of *EphB4* and *EphrinB2* were determined by qRT-PCR analysis in control and serum-deprived WJ-MSCs. *GAPDH* was used as the endogenous control (n = 4). (**b**) WJ-MSCs were cultured for 48 h under serum-deprivation in the presence of 8 μM of EphB4 inhibitor, NVP-BHG712. Total EphB4 and phosphorylated EphB4 (p-EphB4) expression levels were quantified by Western blotting to validate the action of NVP-BHG712 (n = 3). Representative blot images are shown. Band intensities were first quantified relative to GAPDH, which was used as a loading control. Then, the relative expression of p-EphB4 with respect to total EphB4 was calculated and plotted. (**c**) mRNA expression level of *ADAMTS13, VEGF and PDGF*, under serum-deprivation condition in presence of NVP-BHG712 was analysed by qRT-PCR (n = 3). (**d**) Protein expression pattern was detected by Western blotting (n = 3) for the above mentioned markers, representative blot images are shown. (**e**) WJ-MSCs were transfected with *ADAMTS13* or NC siRNA and exposed to serum-deprivation stress for 48 h. Total EphB4 and phosphorylated EphB4 (p-EphB4) expression levels were quantified by Western blotting, and plotted; representative blot images are shown (n = 3). (**f**) WJ-MSCs were treated with NVP-BHG712 for 48 h under serum-deprivation and total ERK and phosphorylated ERK (p-ERK) expression levels were evaluated by Western blotting (n = 3). Representative blot images are shown. Band intensities were first quantified relative to GAPDH, which was used as a loading control. Then, the relative expression of p-ERK with respect to total ERK was calculated and plotted. (**g**) WJ-MSCs were treated with 30 μM of ERK pathway inhibitor, FR180204, for 48 h under serum-deprivation and mRNA expression levels of *ADAMTS13* and angiogenic markers, *VEGF* and *PDGF* were analysed by qRT-PCR (n = 3). (**h**) Protein expression pattern for ADAMTS13, VEGF and PDGF under the same treatment was detected by Western blot analysis and representative blot images are shown (n = 3). Serum-deprived condition has been denoted as no-serum (NS), negative control as NC and control as CT in the figure. Each bar represents mean ± SEM (*p < 0.05, **p < 0.01). Statistical comparisons were assessed using one-way ANOVA followed by Bonferroni’s multiple comparison test. Original blots are presented in Supplementary Fig. S7i–m.

Hence, we wanted to investigate if the EphrinB2/EphB4 signaling regulated the expression of ADAMTS13 and the angiogenic markers in WJ-MSCs. At the mRNA level, an upregulation in the expression levels of both *EphB4* (*p* ~ 0.07) and *EphrinB2* (*p* < 0.05) was observed in WJ-MSCs under serum-deprived condition (Fig. 5a). Next, the action of EphB4 kinase specific inhibitor, NVP-BHG712, was confirmed by reduction in phospho-EphB4 level by Western blotting (*p* < 0.05) (Fig. 5b). On treating serum-deprived WJ-MSCs with NVP-BHG712, a downregulation in the mRNA expression levels of *VEGF* (*p* < 0.01) and *PDGF* (*p* < 0.01) was observed, with no detectable change in the expression of *ADAMTS13,* as compared to untreated serum-deprived WJ-MSCs (Fig. 5c). This was validated at the protein level, where, again, not much change in the expression of ADAMTS13 was noted, but the expression levels of VEGF (*p* < 0.05) and PDGF were downregulated (Fig. 5d). Moreover, when *ADAMTS13* was knocked down in serum-deprived WJ-MSCs by siRNA, phospho-EphB4 level decreased significantly (*p* < 0.05) (Fig. 5e), thus establishing that ADAMTS13 worked upstream of EphrinB2/EphB4 in modulating the expression of VEGF and PDGF.

Previous reports suggested that the MAPK/ERK pathway acted downstream and was activated by EphrinB2/EphB4^21,23^. Next, we assessed the level of phosphorylated ERK1/2 under serum-deprived condition with EphB4 inhibition, and a downregulation (*p* < 0.01) was observed (Fig. 5f). This result indicated that inhibition of the EphrinB2/EphB4 signaling, suppressed the activation of the ERK pathway.

Next, to investigate the role of ERK pathway, if any, in regulating the expression levels of VEGF (*p* < 0.05) and PDGF, we inhibited this pathway and subsequently, a downregulation in the expression of VEGF and PDGF was noted (Fig. 5g,h). Not much change in the expression of ADAMTS13 (Fig. 5g,h), was observed, as was also shown in the pilot study with inhibitors for different signaling pathways (Supplementary Fig. S3). This established that ERK pathway acted upstream of the angiogenic markers, but downstream to ADAMTS13.

These findings overall suggested that ADAMTS13, via the EphrinB2/EphB4 receptor tyrosine kinase axis, activated the ERK pathway, thereby, regulating the expression of the downstream angiogenic markers, VEGF and PDGF.

### Effect of stringent nutrient stress on the expression of ADAMTS13 and the downstream angiogenic markers

In regenerative approaches, MSCs could be transplanted to regions which are marked by a lack of supply of blood thus leading to glucose, nutrient and oxygen deprivation. Glucose can impact cellular survival, functionality and also modulate the activities of certain metabolically relevant pathways^14,24,25^. Certain studies evaluated the effects of glucose-deprivation in combination with serum-deprivation and/or hypoxia on the angiogenic potential of MSCs from other sources^26,27^. Thus, we next evaluated the impact of a more stringent condition of nutrient-deprivation stress, comprising of low glucose in combination with serum-deprivation, on the expression levels of ADAMTS13 and the downstream angiogenic markers, VEGF and PDGF in WJ-MSCs.

**Figure 6:**
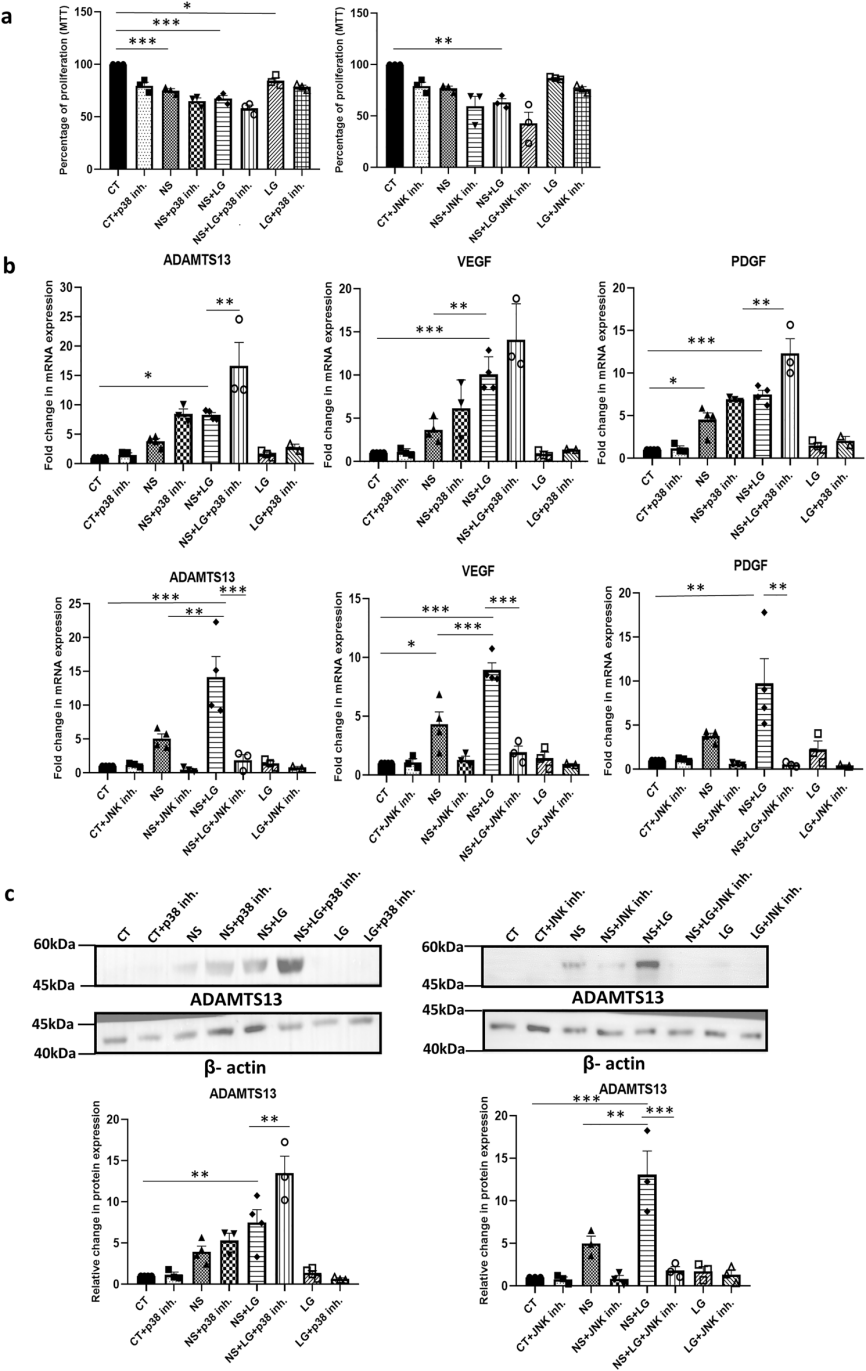

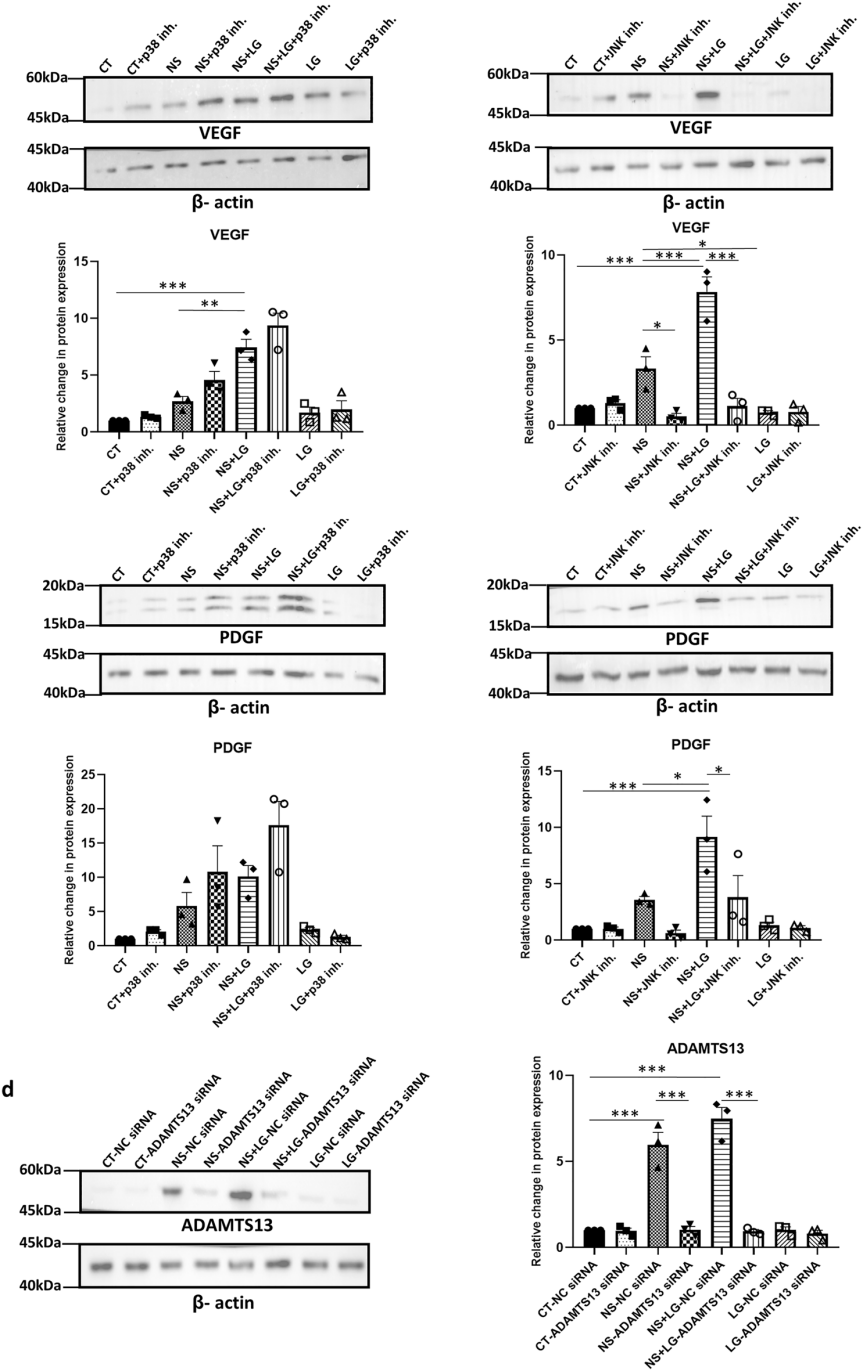

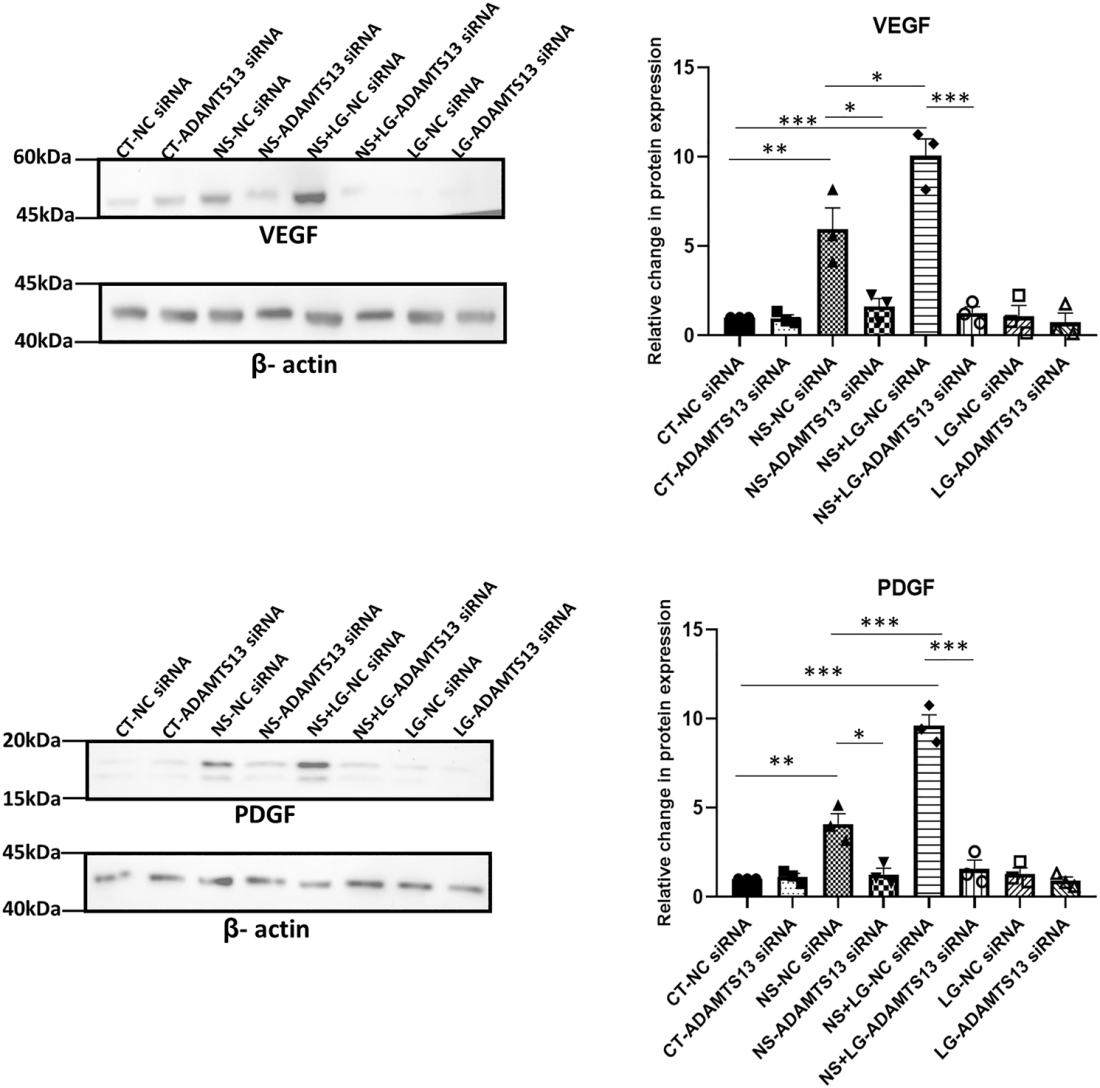
To understand the regulation of angiogenic markers under a more stringent nutrient stress, WJ-MSCs were next exposed to low glucose and serum-deprivation stress, both, in combination and individually, for 48 h. In addition, p38 and JNK pathways were inhibited with 10 μM of SB203580 and 10 μM of SP600125, respectively, under each stress condition. (**a**) MTT assay was performed to compare the percentage of proliferating cells under each of the above conditions (**b**) mRNA expression level of ADAMTS13, VEGF and PDGF was analyzed by qRT-PCR (n ≥ 3). *ꞵ-actin* served as the endogenous control to normalize the gene expression levels. (**c**) Protein expression pattern was detected by Western blotting (n ≥ 3) for the same markers and representative blot images are shown. Band intensities were quantified relative to ꞵ-actin, which was used as a loading control, and plotted. (**d**) WJ-MSCs were transfected with *ADAMTS13* or NC siRNA and exposed to low glucose and serum-deprivation stress, both in combination and individually, for 48 h. Protein expression patterns of ADAMTS13, VEGF and PDGF were detected by Western blotting (n = 3). Representative blot images are shown. Band intensities were quantified relative to ꞵ-actin, which was used as a loading control, and plotted. Serum-deprived condition has been denoted as no-serum (NS), low glucose as LG, combination of no-serum and low glucose as NS + LG, negative control as NC and control as CT in the figure. Each bar represents mean ± SEM (*p < 0.05, **p < 0.01, ***p < 0.001). Statistical comparisons were assessed using one-way ANOVA followed by Bonferroni’s multiple comparison test. Original blots are presented in Supplementary Fig. S7n,o.

The nutrient-deprived WJ-MSCs were characterized for cell surface marker expression by flow cytometry (Supplementary Fig. S1e–h). Next, to test if the viability of MSCs got adversely affected, the population of viable, metabolically active WJ-MSCs, cultured under low glucose and serum-deprivation, individually and in combination, was assessed using MTT assay. The same was also determined with inhibition of the p38 and JNK pathways. A reduction in the percentage of viable, metabolically active cells was observed for the WJ-MSCs cultured under serum-deprived condition (*p* < 0.001) and low glucose condition (*p* < 0.05), individually, as compared to control WJ-MSCs (Fig. 6a). Similarly, on combining low glucose and serum-deprivation stress, a strong reduction in the percent viable cell population was observed as compared to control WJ-MSCs (*p* < 0.001) (Fig. 6a). On inhibiting the p38 or JNK pathways under the combined stress treatment, no further change was observed (Fig. 6b). Next, the mRNA expression levels of *ADAMTS13*, *VEGF* and *PDGF* were analysed under the same conditions. Interestingly, on culturing the WJ-MSCs under the combination stress of low glucose and serum-deprivation, a more pronounced increase in the mRNA expression for each of *ADAMTS13, VEGF* and *PDGF* was noted, as compared to control or serum-deprived conditions (Fig. 6b). It is noteworthy that the low glucose stress alone did not have much of an impact on the expression levels as compared to control or serum-deprived WJ-MSCs. It was further observed, that even under the combination stress condition, the p38 pathway acted as the negative regulator while the JNK pathway acted as the positive regulator for ADAMTS13, with VEGF and PDGF following the same pattern (Fig. 6b). The validation of the same was also carried out at the protein level, which yielded very similar results (Fig. 6c). Further, to affirm its role in modulating the expression of the angiogenic markers even under the combined nutrient stress treatment of serum deprivation and low glucose (Fig. 6d), ADAMTS13 was knocked down using siRNA*.* Concomitantly, a strong downregulation (*p* < 0.001) was noted in the expression of VEGF and PDGF at the protein level. Table 1 summarizes the expression pattern of ADAMTS13, and the angiogenic markers under nutrient stress conditions.

**Table 1:** Summary of the expression patterns of ADAMTS13 and the angiogenic markers under nutrient stress conditions in WJ-MSCs.

Protein	Serum-deprived condition (NS)								Serum-deprived condition in combination with low glucose (NS + LG)			
	NS	+p38 inh	+JNK inh	+Notch inh	+ EphB4 inh	+ERK inh	+p53 siRNA	+ADAMTS13 siRNA	NS + LG	+p38 inh	+JNK inh	+ADAMTS13 siRNA
ADAMTS13	+	++	−	−	+	+	−	−	++	+++	−	−
VEGF	+	++	−	−	−	−	−	−	++	+++	−	−
PDGF	+	++	−	−	−	−	−	−	++	+++	−	−

Serum-deprived condition has been denoted as no-serum (NS) and a combination of serum-deprived and low glucose conditions has been denoted as NS + LG. + denotes an increase in expression as compared to control condition, ++ and +++ denote further increase, while − denotes a decrease in expression as compared to control condition.

It is noteworthy that ꞵ-actin was used as the loading control in place of GAPDH in the low glucose experiments, as GAPDH is a critical component of the glycolytic pathway and its expression was not consistent under low glucose condition. Collectively, these results underlined that subjecting WJ-MSCs to a more stringent nutrient stress condition, led to further upregulation in the expression of ADAMTS13, and the expression of the downstream angiogenic markers.

## Discussion

Post-transplantation, MSCs encounter harsh micro-environmental conditions and get subjected to various forms of stress like nutrient-deprivation, hypoxia, ROS, or an inflammatory-milieu, all of which can hamper their therapeutic potential.

In a previous study from our group, on evaluating the ECM gene expression profile in WJ-MSCs under temperature stress, *ADAMTS13* had shown a considerable increase in expression^28^. An extensive literature review on ADAMTS13 revealed its probable roles in regulating angiogenesis, thrombosis and inflammation^6,29^.

Here we found that the expression of ADAMTS13 was strongly upregulated both at mRNA and protein levels in WJ-MSCs under serum-deprivation stress condition. A possible explanation for the 50 kDa-sized ADAMTS13 in WJ-MSCs could be the existence of an alternatively spliced variant, as reported previously in some human tissues^30,31^. ADAMTS gene expression is often marked by alternative splicing, and this could result in multiple protein products with distinct functional abilities via interacting with ECM, cofactors, and other proteins. Another possibility could be proteolytic cleavage of ADAMTS13, and as reported earlier the distal C terminal of ADAMTS13, containing the CUB domains, could be particularly susceptible to proteolysis by endogenous thrombin^32^.

Additionally, in our study, we also found an upregulation in the expression of some key angiogenic markers, namely, VEGF, PDGF, IL-6 and TNF-α in WJ-MSCs under serum-deprived condition. An interesting point to note here is that another potent angiogenic marker, HGF, was seen to be downregulated under the same conditions. Further, a temporal analysis for 24, 48 and 96 h under serum-deprivation demonstrated a steady increase in expression of ADAMTS13, VEGF and PDGF in a time-dependent manner (Supplementary Fig. S4).

Angiogenesis is a complex phenomenon regulated by a variety of extracellular signals, and many signal transduction pathways. An earlier report showed that p38 negatively regulated angiogenic program in MSCs found in perivascular regions^33^. A study also revealed that hypoxia induced BMSC-derived exosomal HMGB1 promoted angiogenesis via JNK/hypoxia‐inducible factor‐1α signaling^34^. In our case, we found that the JNK pathway acted as a positive regulator for the expression of ADAMTS13, while, the p38 pathway negatively regulated it in WJ-MSCs cultured under serum-deprivation stress. Correspondingly, the key angiogenic markers, also followed the same pattern of expression as ADAMTS13. Furthermore, we found that the Notch signaling pathway, which is reported to be playing critical roles in the maturation and sprouting of blood vessels, was also acting as a positive regulator of ADAMTS13 expression in WJ-MSCs. In addition, p53, a cellular stress marker, was upregulated under serum-deprivation stress and knocking it down led to a reduction in the expression of ADAMTS13 along with a reversal in the expression of the key angiogenic markers. Hence, the possibility of p53 regulating the expression of ADAMTS13, as a transcription factor, could exist.

Though our findings demonstrated the involvement of p53, JNK, p38 and Notch pathways, individually, in regulating the expression of ADAMTS13, the knowledge if they act in parallel or interact with each other is lacking in this study. Further experiments would be needed to evaluate the same.

Knocking down of ADAMTS13 led to reversal in the expression of the key angiogenic markers, further confirming that ADAMTS13 could influence the angiogenic potential of WJ-MSCs. This observation corroborated with the findings of a previous report where knockdown of ADAMTS13 in HTR-8/SVNEO, a trophoblast cell line, significantly brought down their angiogenic potential, by resulting in reduced levels of tube formation in Matrigel assay^35^. Moreover, addition of recombinant human ADAMTS13 (full-length) to the *ADAMTS13* siRNA transfected WJ-MSCs, had a rescue effect in the expression of the key angiogenic markers (Supplementary Fig. S5).

EphrinB2/EphB4 signaling influences multiple pathways, including angiogenesis, and MSCs derived from bone marrow, umbilical cord blood, adipose tissue have been shown to express many of the Ephrin/Eph molecules^36^. To the best of our knowledge, for the first time, we demonstrated the expression of EphrinB2/EphB4 molecules in WJ-MSCs and its inhibition leading to downregulation in the expression of VEGF and PDGF in serum-deprived WJ-MSCs. Although HGF showed an opposite trend in expression compared to other angiogenic markers studied here, the impact of EphrinB2/EphB4 signaling on its expression was not found to be conclusive. Further studies would be needed to understand what regulated HGF in WJ-MSCs under nutrient stress conditions.

MSCs are often transplanted in ischemic regions which are marked by deprivation of serum, glucose, oxygen and other nutrients. To assess the molecular mechanism of angiogenesis under a more stringent nutrient stress to mimic an ischemia-like condition, we next challenged the WJ-MSCs with serum-deprivation and low glucose, in combination. A reduction in metabolically active cell percentage was noted under both the conditions in combination, and individually. A cell cycle phase analysis using PI staining followed by flow cytometry confirmed an increase in the percentage of G0/G1 population with a corresponding decline in S phase and G2/M phase cells in both, serum-deprived and serum-deprived combined with low glucose treated WJ-MSCs, indicating a G0/G1 arrest (Supplementary Fig. S6a,b).

Markedly higher expression levels of ADAMTS13, VEGF and PDGF were observed as compared to those under serum-deprivation state alone. Though low glucose alone did not have much of an effect on their expression levels, but in the presence of serum deprivation, a heightened response was noted.

Additionally, the stronger adhesion exhibited by WJ-MSCs under serum-deprivation condition got reversed on knocking down ADAMTS13. Hence, alongside modulating the expression of angiogenic markers, ADAMTS13’s role in altering cell–matrix adhesion was also established in WJ-MSCs under serum-deprived condition.

## Conclusion

**Figure 7:**
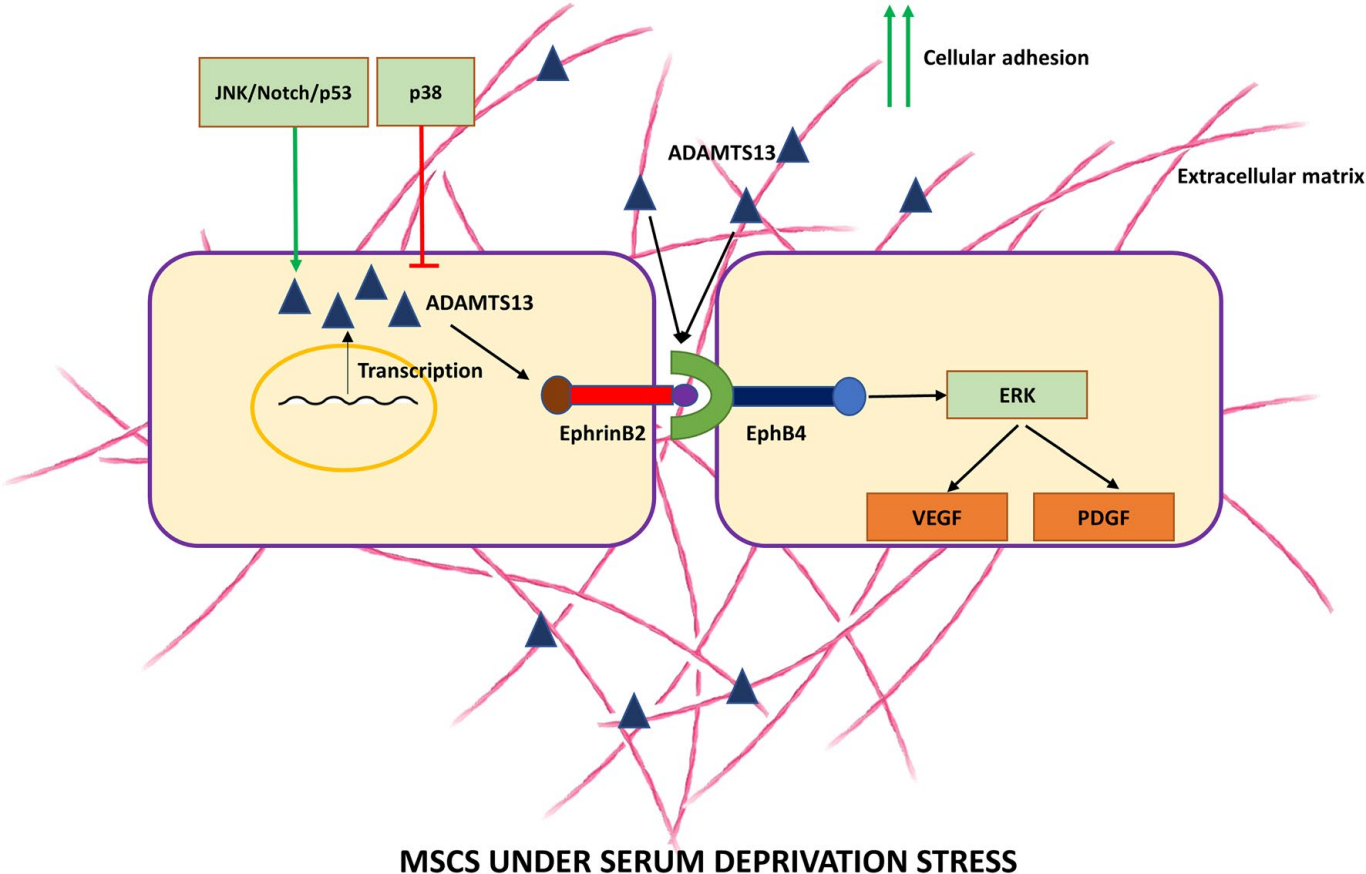
The expression of ADAMTS13 was seen to be upregulated in WJ-MSCs under serum-deprived stress. JNK and p38 signaling pathways acted as positive and negative regulators of ADAMTS13 expression, respectively. Additionally, Notch pathway and p53 were indicated to regulate its expression. The increased ADAMTS13 level modulated the expression of some key angiogenic markers via the EphrinB2/EphB4 signaling and ERK pathway acting downstream. It also impacted cellular adhesion of WJ-MSCs under serum-deprived condition.

To conclude, we have established the role of the matrix metalloproteinase, ADAMTS13, in regulating the expression of some key angiogenic markers in WJ-MSCs under serum deprivation stress, via the EphrinB2/EphB4 axis (Fig. 7). To the best of our knowledge, this is also the first report elucidating the underlying molecular signaling pathways involved in the regulation of ADAMTS13 in MSCs under nutrient-deprivation stress. Overall, our observations and findings lay the foundation for furthering the prospects of ADAMTS13-based MSC therapy in treating myocardial infarction and other ischemic disorders.

## Methods

### Cell culture

Collection of human umbilical cords (n ~ 9) was done after full-term births (vaginal or cesarean delivery) with informed consent from the donor, following the guidelines laid down by the Institutional Ethics Committee (IEC) and Institutional Committee for Stem Research Cell and Therapy (IC-SCRT) at IISER Kolkata, India. All methods were performed in accordance with the guidelines and regulations of the Declaration of Helsinki.

MSCs were isolated from the perivascular region of the umbilical cords, also known as the Wharton’s Jelly (WJ), by explant culture method, as described previously^37^. On reaching 70–80% confluency, WJ-MSCs were dislodged from the tissue culture dishes with TrypLE Express (Thermo Fisher Scientific, Waltham, MA, USA), which is a gentle, animal origin-free recombinant enzyme. The WJ-MSCs plated between passages four to six at a seeding density of 5000 cells/cm^2^ were used for the experiments. They were cultured in complete medium consisting of KnockOut DMEM (Dulbecco’s modified Eagle’s medium, containing 4 g/l of glucose) supplemented with 10% fetal bovine serum (FBS), 2 mM l-glutamine and 1X PenStrep (all from Thermo Fisher Scientific). After 24 h, the serum-deprivation stress was induced by culturing the cells in KnockOut DMEM Medium supplemented with 2 mM glutamine and 1X PenStrep, but without any serum for the next 48 h. Similarly, the low glucose stress was induced by culturing the cells in DMEM, low glucose, pyruvate medium, containing 1 g/l of glucose, (Thermo Fisher Scientific) supplemented with 10% FBS and 1X PenStrep for the next 48 h. Additionally, a more stringent nutrient-deprivation stress was induced by culturing the cells in serum-deprived, low glucose medium for 48 h. Meanwhile, control cells were grown in complete medium for a total duration of 72 h. For the different signaling pathway inhibition experiments, WJ-MSCs were treated with specific small molecule inhibitors and their respective vehicle controls under serum-deprived and low glucose condition for a period of 48 h. The inhibitors for JNK pathway, SP600125, (Sigma-Aldrich, Saint Louis, MO, USA); p38 pathway, SB203580, (Santa Cruz Biotechnology, Dallas, TX, USA); ERK pathway, FR180204 (Sigma-Aldrich); PI3K pathway, LY294002 (Sigma-Aldrich); NF- κꞵ pathway, BAY 11-7082 (Sigma-Aldrich), Notch pathway, DAPT (GSI-IX, LY-374973), (Selleckchem, Houston, TX, USA) and EphB4, NVP-BHG712 (MedChemExpress, Princeton, NJ, USA) were added at optimized concentrations of 10 μM, 10 μM, 30 μM, 20 μM, 4 μM, 20 μM and 8 μM respectively.

For temporal expression studies, WJ-MSCs were initially plated under standard culture conditions for 24 h. After this, the cells were subjected to serum-deprivation stress for 24, 48 and 96 h, respectively.

### Characterization of WJ-MSCs by immunophenotyping

For immunophenotyping of WJ-MSCs, cells at passage 4–5 were harvested in ice-cold phosphate-buffered saline (PBS) and 1 × 10^5^ cells were aliquoted in each flow-cytometry tube. The WJ-MSCs were labelled with phycoerythrin (PE)-conjugated anti-human CD73, CD90, CD105 and CD34 (all from BD Pharmingen, San Jose, CA, USA) and incubated in 4 °C in dark for 1 h. Following this, the cells were resuspended in 500 μl PBS and analysed by flow cytometry using BD LSRFortessa™ Cell Analyzer (BD Biosciences, San Jose, CA, USA). The respective mouse isotype antibodies were used as controls.

### Characterization of WJ-MSCs by in-vitro differentiation

The in-vitro differentiation potential of WJ-MSCs was assessed by evaluating their ability to differentiate into adipogenic and osteogenic lineages. At passages 4–5, confluent monolayer cultures of WJ-MSCs were induced with StemPro^®^ Adipogenesis Differentiation Kit and StemPro^®^ Osteogenesis Differentiation Kit, respectively, (both from Thermo Fisher Scientific) and maintained in culture as per manufacturer’s protocol. Differentiated cells were stained with Oil Red O (Sigma-Aldrich) after 16–18 days (for adipogenic differentiation) and Alizarin Red S (Sigma-Aldrich) after 21 days (for osteogenic differentiation). Corresponding uninduced cultures served as controls. Images were captured using Olympus BX51 (Olympus, Shinjuku, Tokyo, Japan) microscope.

### Cell morphology

Cell morphology images were captured using Eclipse TS100 inverted phase-contrast microscope (Nikon, Minato City, Tokyo, Japan). Morphology was assessed and quantified by measuring circularity index (CI) using ImageJ (NIH) software. The formula used was CI: 4π(area)/(perimeter)^2^, where CI denotes a range of value from 0 to 1 as 1 corresponds to a rounded cell morphology while values close to 0 represents an elongated cell shape.

### Growth kinetics

Population doublings were estimated using the formula: X = [log10(NH)-log10(NI)]/log10(2). The population doubling time was obtained by the formula: TD = *t* ∗ log10(2)/(log10(NH) − log10(NI). NI: the inoculum cell number; NH: the cell harvest number, and *t* is the time of the culture (in hours).

### RNA isolation and cDNA synthesis

Total RNA was isolated using RNAiso Plus (Takara, Kusatsu, Shiga, Japan) as per the manufacturer’s protocol and the RNA yield was quantified using Nanodrop 2000 spectrophotometer (Thermo Fisher Scientific). cDNA synthesis was performed using Verso cDNA synthesis kit (Thermo Fisher Scientific), as per the manufacturer’s protocol.

### Quantitative reverse-transcription polymerase chain reaction

Quantification of the mRNA expression was done by performing qRT-PCR. PowerUp SYBRGreen Master Mix (Applied Biosystems, Waltham, MA, USA) and Bio-Rad CFX96 Real Time System (Bio-Rad, Hercules, CA, USA) were used. The fold changes in mRNA expression were quantified by 2^−ΔΔCT^ method. *GAPDH* and *ꞵ-actin* were considered as the endogenous control genes. The primer sequences are listed in Table 2.

**Table 2:** Primer sequences used for qRT-PCR.

Sl. no	Gene name	Forward primer (5′–3′)	Reverse primer (5′–3′)
1	*ADAMTS13*	CACAGGCCGTGTCTTCTTACTTGA	TCAGGCTCTGTCAGAATGACCATC
2	*VEGF*	TTGCCTTGCTGCTCTACCTCCA	GATGGCAGTAGCTGCGCTGATA
3	*PDGF*	CAGCGACTCCTGGAGATAGACT	CGATGCTTCTCTTCCTCCGAATG
4	*HGF*	ACGAACACAGCTTTTTGCCTT	GCAAGAATTTGTGCCGGTGT
5	*IL-6*	ATGAACTCCTTCTCCACAAGC	GTTTTCTGCCAGTGCCTCTTTG
6	*TNF-α*	CTCTTCTGCCTGCTGCACTTTG	ATGGGCTACAGGCTTGTCACTC
7	*p53*	GAGCTGAATGAGGCCTTGGA	CTGAGTCAGGCCCTTCTGTCTT
8	*EphB4*	ACATCACAGCCAGACCCAACTG	AGGCAGAGAACTGCGACCACAA
9	*EphrinB2*	GCAAGTTCTGCTGGATCAACCAG	GCTGTTGCCGTCTGTGCTAGAA
10	*GAPDH*	GAGTCAACGGATTTGGTCGT	TTGATTTTGGAGGGATCTCG
11	*β-actin*	CAACTGGGACGACATGGAGAAA	GATAGCAACGTACATGGCTGGG

### Western blotting

WJ-MSCs, cultured under different conditions, were lysed using RIPA lysis buffer containing protease inhibitor cocktail, PMSF and phosphatase inhibitor cocktail (all from Santa Cruz Biotechnology). The lysates were then centrifuged at 8000 rpm for 10 min at 4 °C and the supernatants were collected.

The conditioned medium collected from the WJ-MSCs was centrifuged, lyophilized and resuspended in appropriate amount of basal medium. The concentration of protein in cell lysates as well as conditioned media was quantified by Bradford assay (Bio-Rad). The samples were then separated by SDS PAGE using 8%, 10% or 15% polyacrylamide gel and were subsequently transferred to polyvinylidene difluoride (PVDF) membrane (Merck Millipore, Burlington, MA, USA). Membranes were blocked using 5% skimmed milk or 5% bovine serum albumin (BSA) and incubated with the respective primary antibodies overnight at 4 °C. The primary antibodies used were anti-ADAMTS13 (MAB4245, R&D Systems, Minneapolis, MN, USA/ DF9171, Affinity Biosciences, Cincinnati, OH, USA) at 1:1000 dilution, anti-p53 (sc-126, Santa Cruz Biotechnology) at 1:2500 dilution, anti-VEGF (MAB293, R&D Systems) at 1:2000 dilution, anti-PDGF-B (NB110-60980, Novus Biologicals, Littleton, CA, USA) at 1:1000 dilution, anti-HGF-β (52445T, Cell Signaling Technology, Danvers, MA, USA) at 1:2000 dilution, anti-p44/p42 MAPK (Erk1/2) (9102S, Cell Signaling Technology) at 1:3000 dilution, anti-phospho-p44/p42 MAPK (Erk1/2) (9101S, Cell Signaling Technology) at 1:2500 dilution, anti-EphB4 (14960S, Cell Signaling Technology) at 1:1000 dilution, anti-phospho-EphB4 (Tyr987) (PA5-64792, Thermo Fisher Scientific) at 1:1000 dilution, anti-GAPDH (sc-47724, Santa Cruz Biotechnology) at 1:5000 dilution and anti-β-actin (4970T, Cell Signaling Technology) at 1:3000 dilution. After overnight incubation with primary antibody, the membranes were incubated with the respective secondary antibody at room temperature for 1.5 h. The secondary antibody used was HRP-linked anti-mouse IgG (7076P2, Cell Signaling Technology) or HRP-linked anti-rabbit IgG (sc-2357, Santa Cruz Biotechnology); both at 1:5000 dilution. The bands were detected by enhanced chemiluminescence. Signals were captured by the G:BOX gel imaging system (Syngene, Frederick, MD, USA) and the band intensities were determined using GeneSys image acquisition software (Syngene).

### siRNA transfection

WJ-MSCs were grown till 50–60% confluency. Transfection was done by using Lipofectamine 3000 in Opti-MEM I (both from Thermo Fisher Scientific) medium as per manufacturer’s protocol. siRNAs generated against *ADAMTS13* (Thermo Fisher Scientific) and *p53* (Santa Cruz Biotechnology) were used at concentrations of 100 nM and 30 nM respectively. For negative control (NC) MISSION^®^ siRNA universal negative control (Sigma-Aldrich) was used. After 18–20 h post transfection, WJ-MSCs were subjected to serum-deprivation stress for the next 36–48 h and harvested.

### Treatment with rhADAMTS13

Post-transfection with siRNA generated against *ADAMTS13,* WJ-MSCs were subjected to serum-deprivation stress, with the addition of 300 ng/ml of recombinant human ADAMTS13 (full-length) (R&D Systems). After 36–48 h, the cells were harvested and analyzed for the expression of ADAMTS13 and the key angiogenic markers.

### De-adhesion dynamics analysis

Cell de-adhesion dynamics was assessed by performing live cell time lapse imaging. Cells were treated with 0.5 mM EDTA (Sigma-Aldrich), followed by live cell imaging using Olympus IX81 (Olympus) microscope to capture de-adhesion of the cells from the surface. Change in cell spread area over time was calculated and the normalized cell spread area was plotted against time. The data was fitted in Boltzmann’s equation, from which the two time constants, τ1 and τ2 were obtained^38^.

### Immunofluorescence staining

For immunofluorescence staining, cells were grown on coverslips and the cover-slips were briefly fixed in 4% paraformaldehyde in PBS for 20 min at room temperature. Subsequently, they were permeabilized with 0.1% Triton-X-100 in PBS for 10 min at room temperature. After blocking with 3% BSA in PBS for 1 h, the cells were incubated with the primary antibody, anti-ADAMTS13 (R&D Systems) at 1:50 dilution, overnight at 4^◦^C. After overnight incubation, the coverslips were incubated with the secondary antibody, goat anti-mouse IgG H&L (Alexa Fluor^®^ 488) (A11001, Thermo Fisher Scientific), in dark, at room temperature for 1 h at 1:500 dilution. DAPI (Sigma-Aldrich) was used to stain the nucleus at 1:5000 dilution. The coverslips were mounted using VECTASHIELD^®^ (Vector Laboratories, Newark, CA, USA) as anti-fade mounting medium. In order to rule out any false signal due to non-specific binding by the secondary antibody, a sample where the addition of the primary antibody was omitted, but the secondary antibody was added, served as a control. The cell images were captured at room temperature using ZEN software (Zeiss, Oberkochen, Germany) at 40X magnification in Zeiss Apotome module microscope. The mean fluorescence intensities of expression were quantified using ImageJ (NIH) Software.

### MTT assay

To evaluate cell proliferation, 3-(4,5-dimethylthiazol-2-yl)-2,5-diphenyltetrazolium bromide (MTT) assay was performed using standard protocol. WJ-MSCs were seeded onto a 96-well plate and subjected to different treatment conditions. Next, MTT (Sigma–Aldrich) dye was added to each well at a final concentration of 0.5 mg/ml and incubated at 37 °C. The reaction was stopped by adding dimethyl sulfoxide (DMSO; Merck, Rahway, NJ, USA) to solubilize purple-coloured formazan crystals, and again incubated at 37 °C for 20 min. After this, absorbance was measured at 595 nm using a plate reader, BioTek Epoch (Agilent, Santa Clara, CA, USA). The percentage changes in proliferation were calculated with respect to control after subtracting the background absorbance. Each assay was carried out in quadruplets.

### Cell cycle analysis

To analyze cells in different phases of the cell cycle, control and serum-deprived/low glucose cells (both individually and in combination) were harvested, resuspended in PBS and fixed in 70% ethanol in PBS. The cell suspensions were then kept overnight, at − 20 °C. The next day, the cells in suspension were pelleted down and incubated with a mixture of 50 μg/ml propidium iodide (PI) (Sigma-Aldrich) and 100 µg/ml RNAse A (Thermo Fisher Scientific) at 37 °C for 20–30 min. After incubation, samples were analyzed by BD LSRFortessa™ Cell Analyzer (BD Biosciences), and the percentages of cells in different phases of the cell cycle were analyzed using BD FACSDiva ™ software (BD Biosciences).

### Statistical analysis

Normally distributed samples with n ≥ 3 were used for most experiments, where n represents biological replicates. All data are presented as mean ± standard error of the mean (SEM). Data analysis and graphical representations were performed using GraphPad Prism5/8 software (GraphPad, La Jolla, CA, USA). Statistical comparisons were assessed using column statistics (one sample t test), two-tailed Student’s t-test, or one-way ANOVA followed by Bonferroni’s multiple comparison test. Significance was accepted at *p* ≤ 0.05.

### Supplementary Information

Supplementary Figures.

## Data Availability

The datasets used and analyzed in the study are available from the corresponding author upon reasonable request.
