# Supplementary material for: ADAMTS13 regulates angiogenic markers via Ephrin/Eph signaling in human mesenchymal stem cells under serum-deprivation stress
Source: Sci Rep. 2024 Jan 4;14:560. doi: 10.1038/s41598-023-51079-z (PMC10766954; doi:10.1038/s41598-023-51079-z)
Supplement: Supplementary file 1 — Supplementary Figures. [file 41598_2023_51079_MOESM1_ESM.pdf]

<u>Supplementary Information</u>

ADAMTS13 Regulates Angiogenic Markers via Ephrin/Eph Signaling in Human Mesenchymal Stem Cells under Serum-deprivation Stress

Srishti Dutta Gupta and Malancha Ta*.

Indian Institute of Science Education and Research, Kolkata (IISER Kolkata), India.

Srishti Dutta Gupta, Email: sdg19rs080@iiserkol.ac.in
Malancha Ta, Email: malancha.ta@iiserkol.ac.in

Supplementary Figure S1

a

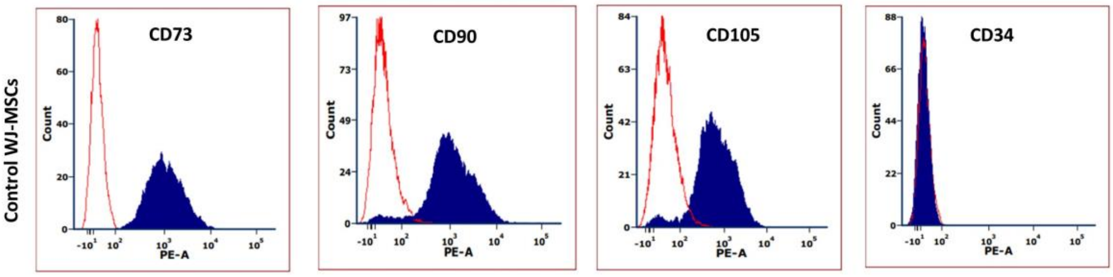

b

Sl. No	CD marker	Percentage±SEM
1.	CD73	98.46±0.28
2.	CD90	99.31±0.14
3.	CD105	97.48±0.35
4.	CD34	0.15±0.02

c

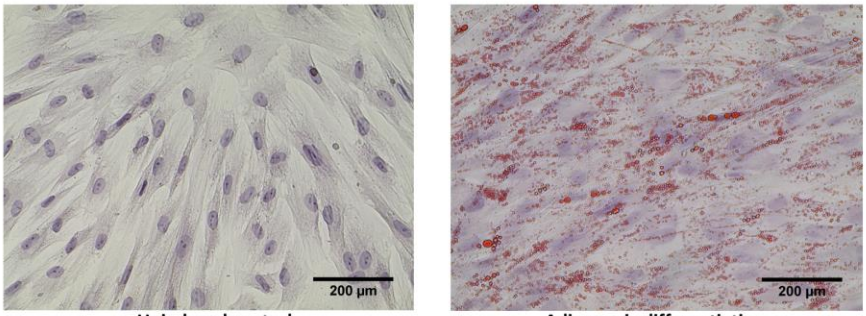

Uninduced control　　Adipogenic differentiation

d

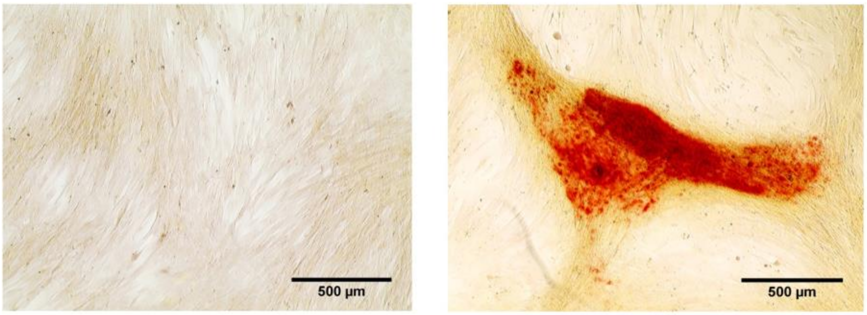

Uninduced control　　Osteogenic differentiation

e

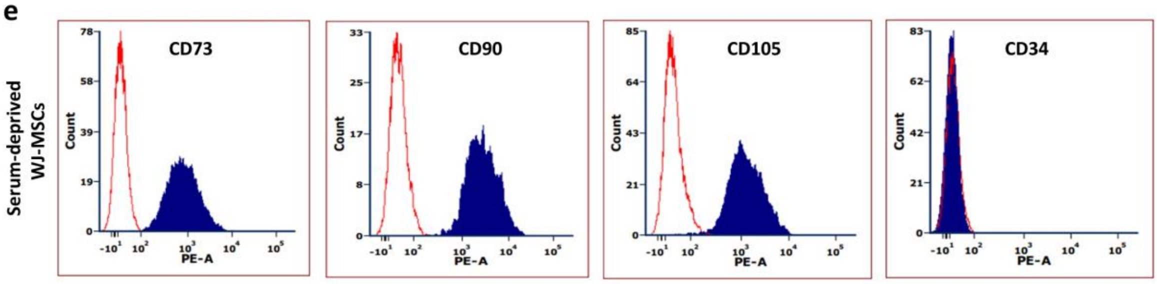

f

Sl. No	CD marker	Percentage±SEM
1.	CD73	98.40±0.49
2.	CD90	99.27±0.37
3.	CD105	98.77±0.23
4.	CD34	0.17±0.07

g

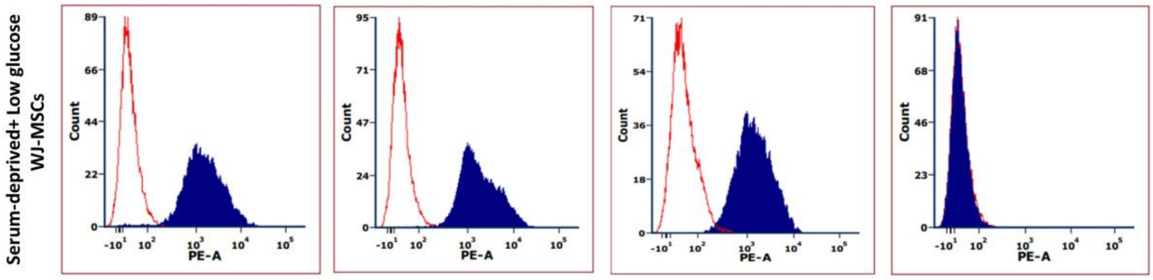

h

Sl. No	CD marker	Percentage±SEM
1.	CD73	97.60±0.20
2.	CD90	99.10±0.11
3.	CD105	98.70±0.47
4.	CD34	0.26±0.08

Characterization of WJ-MSCs (**a**) Representative histograms showing immunophenotypic analysis of WJ-MSCs as assessed by flow cytometry. They were demonstrated to be positive for CD73, CD90 and CD105; and negative for CD34 (n=9). Background signals are depicted by open histograms, while positive reactivity with the indicated antibodies are depicted by shaded histograms. (**b**) Table representing the percentage±SEM of WJ-MSCs expressing CD73, CD90, CD105 and

CD34 (n=9). (**c**) To demonstrate the differentiation potential of WJ-MSCs, they were induced to undergo adipogenic differentiation and stained with Oil Red O stain. Representative adipogenic differentiation images showing the presence of red oil droplets are displayed. The corresponding uninduced control culture did not show any staining. 40X magnification. Scale bar: 200 μm (n=3) (**d**) WJ-MSCs were induced for osteogenic differentiation and stained with Alizarin Red S stain. Representative differentiation images showing calcium depositions (stained red) are displayed. The corresponding uninduced control culture did not show any staining. 10X magnification. Scale bar: 500 μm (n=3). (**e**) Representative histograms showing immunophenotypic analysis of WJ-MSCs, cultured under serum-deprived condition, as assessed by flow cytometry. They were demonstrated to be positive for CD73, CD90 and CD105; and negative for CD34 (n=3). Background signals are depicted by open histograms and positive reactivity with the antibodies are depicted by shaded histograms. (**f**) Table representing the percentage±SEM of WJ-MSCs expressing CD73, CD90, CD105 and CD34, under serum-deprived condition (n=3). (**g**) Representative histograms showing immunophenotypic analysis of WJ-MSCs, cultured under a combined nutrient stress consisting of low glucose and serum-deprivation, as assessed by flow cytometry. They were demonstrated to be positive for CD73, CD90 and CD105; and negative for CD34 (n=3). Background signals are depicted by open histograms and positive reactivity with the antibodies are depicted by shaded histograms. (**h**) Table representing the percentage±SEM of WJ-MSCs expressing CD73, CD90, CD105 and CD34, under the combined nutrient stress (n=3).

Supplementary Figure S2

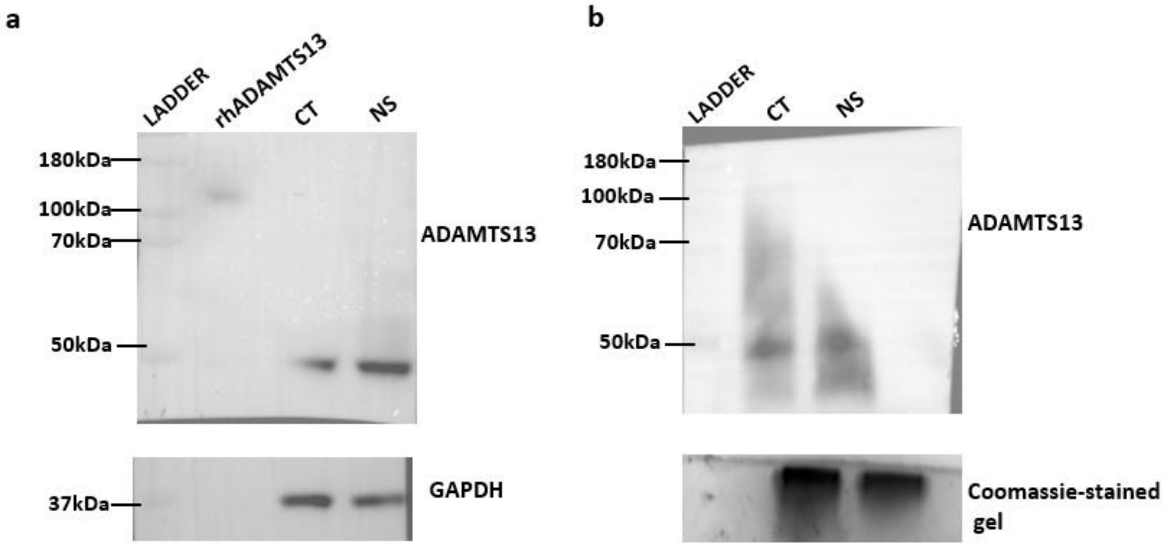

ADAMTS13 protein band size (a) Western blot analysis was performed to compare the ADAMTS13 protein size between recombinant human ADAMTS13 (full-length) and control and serum-deprived WJ-MSC cell lysates. A band of ~150kDa was observed for full-length recombinant human ADAMTS13 whereas cell lysates from WJ-MSCs demonstrated a band of ~50kDa. (b) Native PAGE under non-denaturing conditions was also performed to confirm the presence of ADAMTS13 at ~50kDa in WJ-MSC samples. Serum-deprived condition has been denoted as no-serum (NS), control as CT and recombinant human ADAMTS13 as rhADAMTS13 in the figures.

Supplementary Figure S3

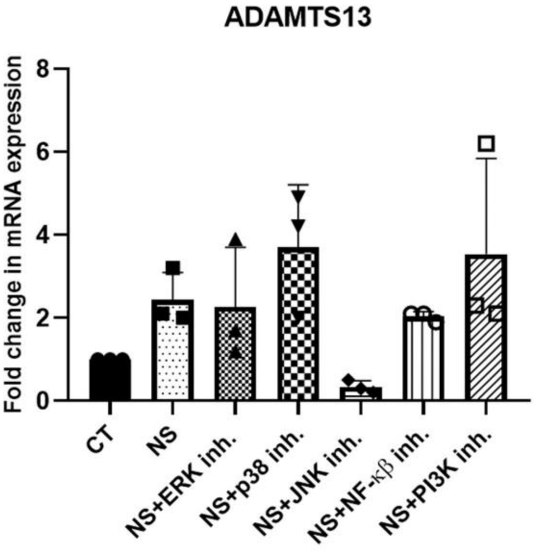

A pilot study to explore regulation of *ADAMTS13* at the molecular level by inhibiting ERK, p38, JNK, NF-κβ and PI3Ksignaling pathways with specific small molecule inhibitors under serum-deprived condition in WJ-MSCs, as demonstrated by qRT-PCR (n=3). *GAPDH* was used as an endogenous control to normalise the gene expression levels. Serum-deprived condition has been denoted as no-serum (NS) and control as CT in the figure. Each bar represents mean±SEM. Statistical comparisons were assessed using one-way ANOVA followed by Bonferroni's multiple comparison test.

Supplementary Figure S4

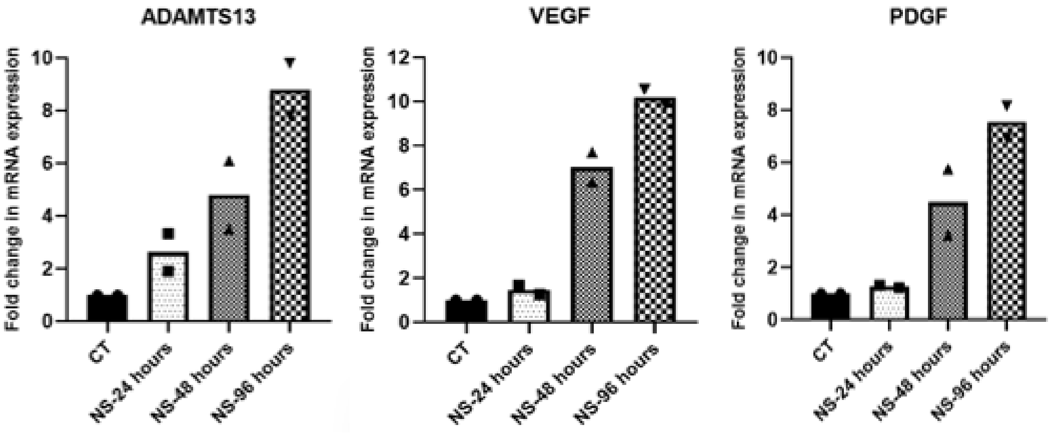

A temporal analysis of the expression patterns of *ADAMTS13*, *VEGF* and *PDGF* in WJ-MSCs when exposed to serum-deprivation stress over an increasing time period of 24, 48 and 96 hours, respectively, as demonstrated by qRT-PCR (n=2). *GAPDH* was used as an endogenous control to normalise the gene expression levels. Serum-deprived condition has been denoted as no-serum (NS) and control as CT in the figure.

Supplementary Figure S5

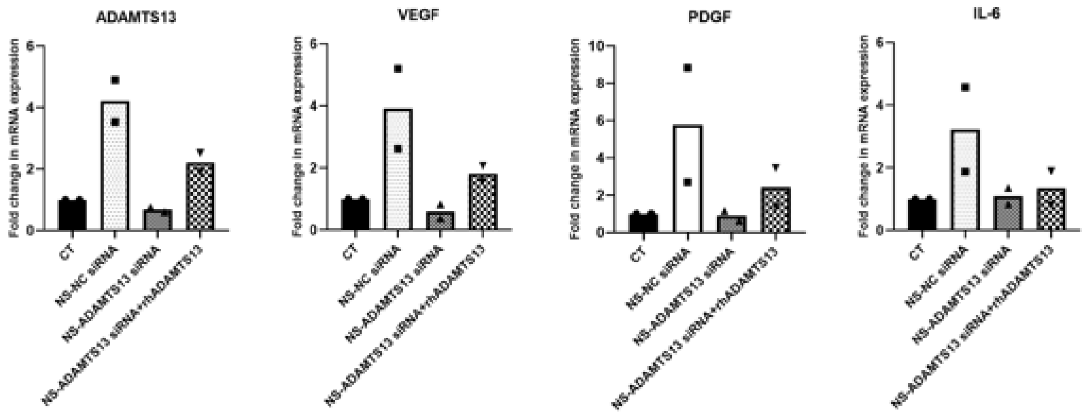

To explore the impact of addition of recombinant human ADAMTS13 (full-length) on the expression patterns of *VEGF*, *PDGF* and *IL-6* in *ADAMTS13* siRNA transfected WJ-MSCs under serum-deprivation condition, as demonstrated by qRT-PCR (n=2). *GAPDH* was used as an endogenous control to normalise the gene expression levels. Serum-deprived condition has been denoted as no-serum (NS), negative control as NC, control as CT and recombinant human ADAMTS13 as rhADAMTS13 in the figure.

Supplementary Figure S6

a

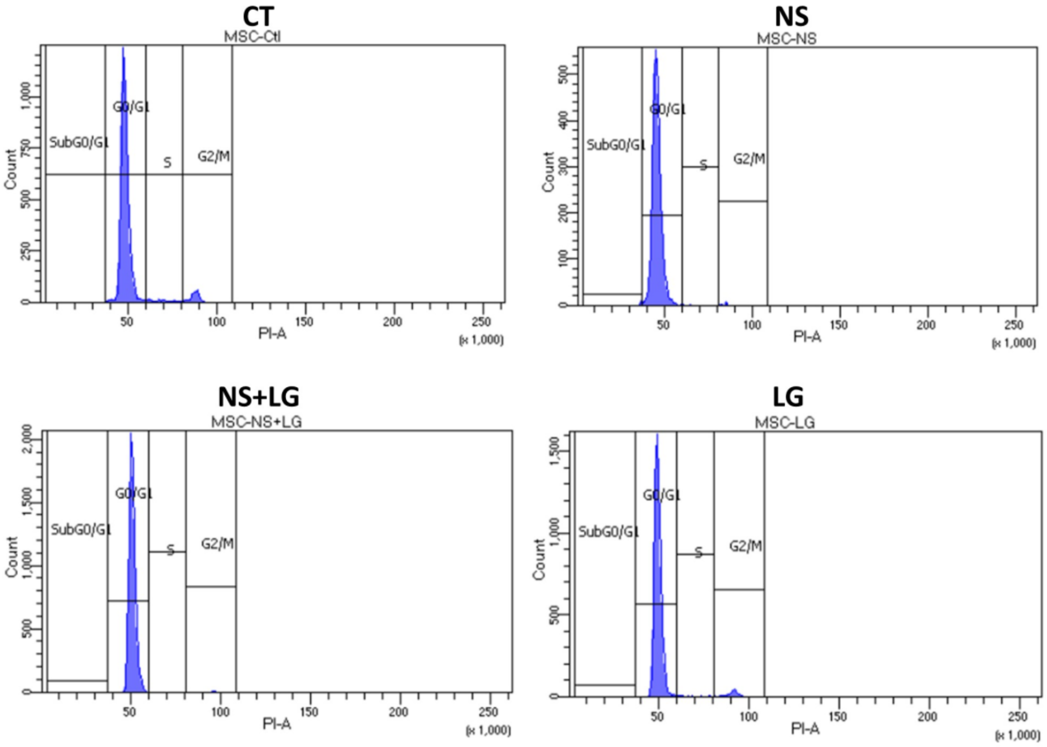

b

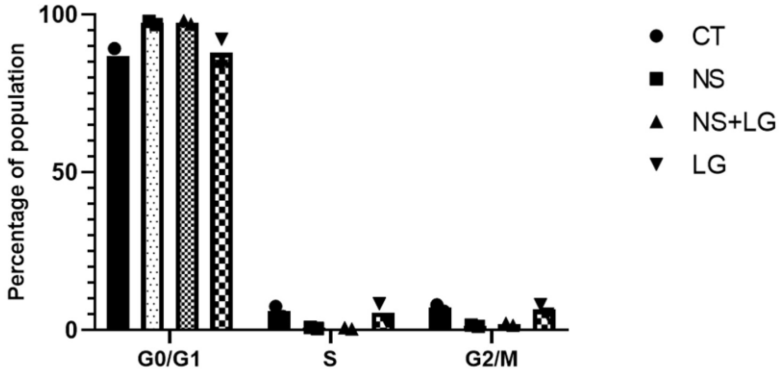

Cell cycle analysis of WJ-MSCs. (**a**) Control and nutrient-deprived WJ-MSCs were stained with propidium iodide, and analysed by flow cytometry to evaluate the percentage of cells present in different phases of the cell cycle. Representative cell cycle analysis data are shown (n=2). (**b**) Percentages of cells in each phase of the cell cycle are also represented by histograms (n=2). Serum-deprived condition has been

denoted as no-serum (NS), low glucose as LG, combination of no-serum and low glucose as NS+LG and control as CT in the figure.

Supplementary Figure S7

Full-length blot/gel images, that have been used in Figures 1-6.

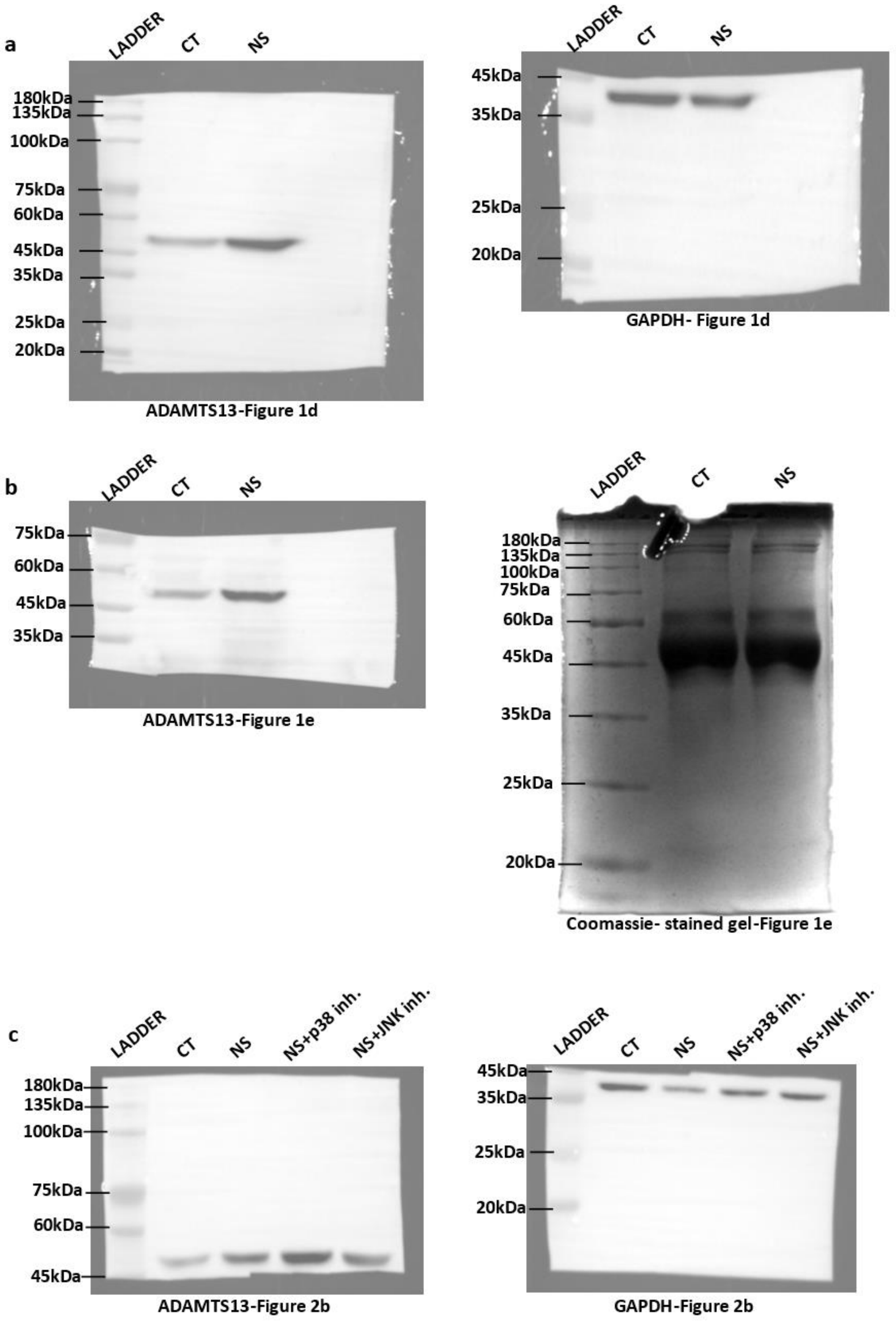

d

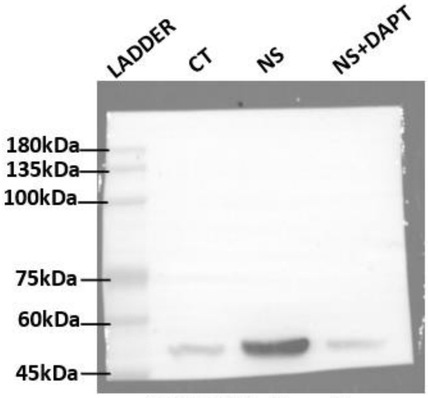

ADAMTS13-Figure 3b

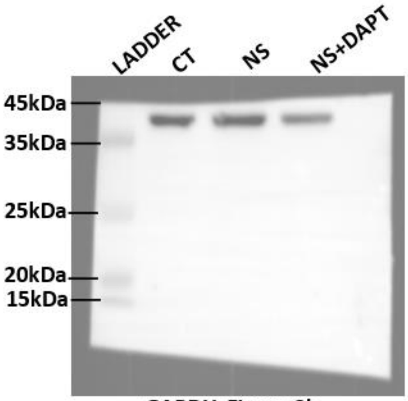

GAPDH-Figure 3b

e

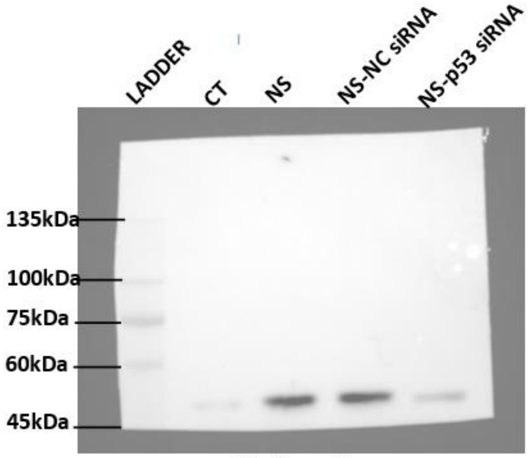

p53-Figure 3e

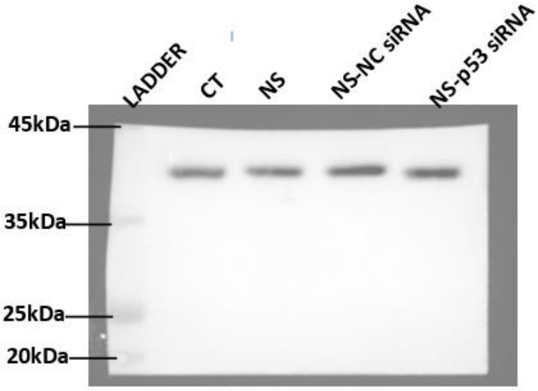

GAPDH-Figure 3e

f

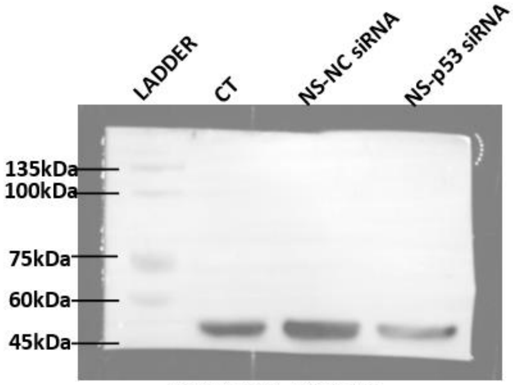

ADAMTS13-Figure 3g

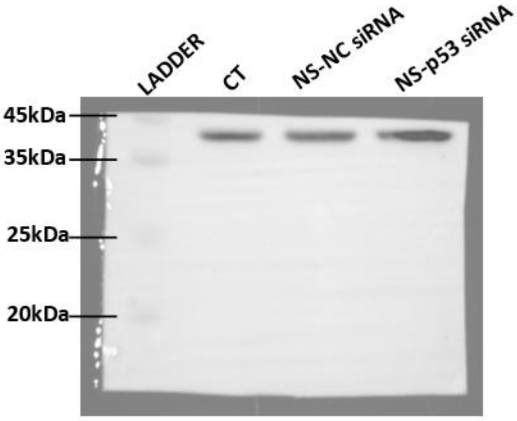

GAPDH-Figure 3g

g

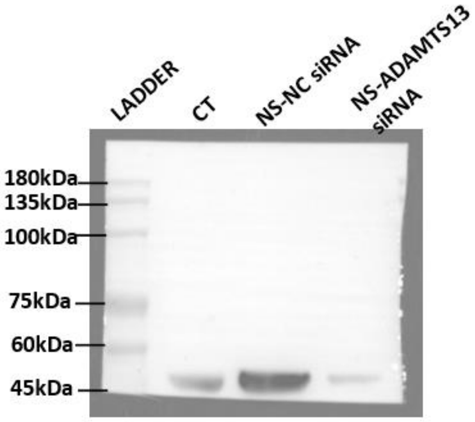

ADAMTS13-Figure 4b

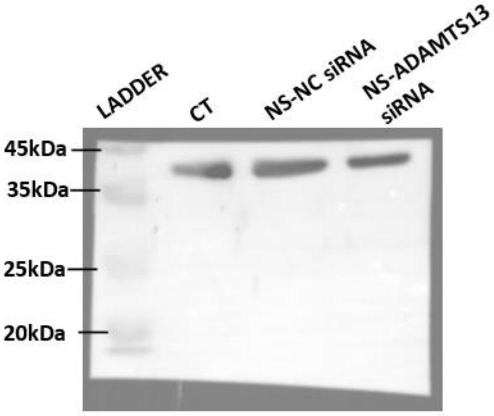

GAPDH-Figure 4b

h

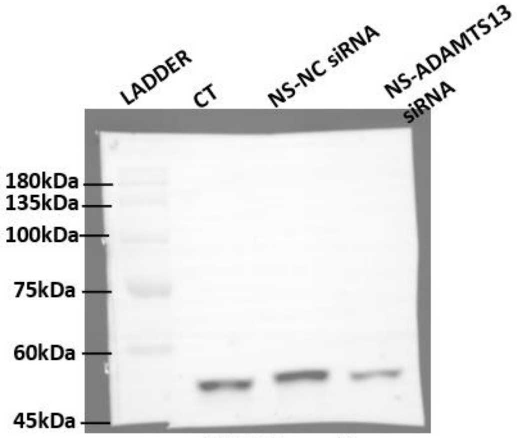

VEGF-Figure 4i

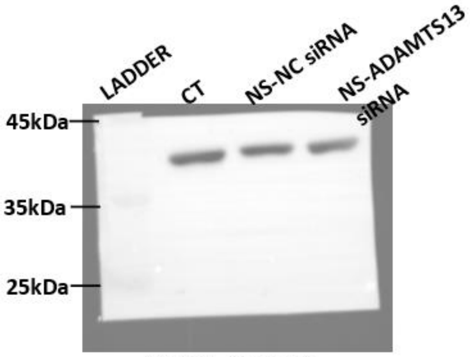

GAPDH-Figure 4i

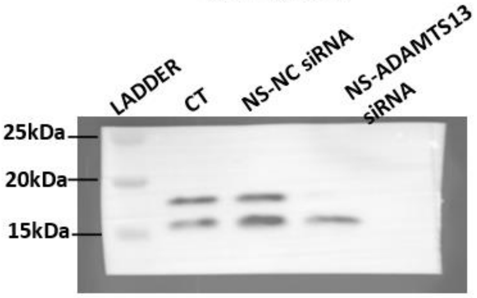

PDGF-Figure 4i

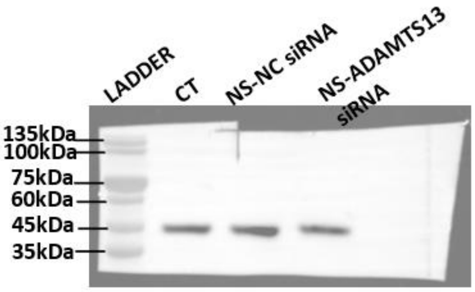

GAPDH-Figure 4i

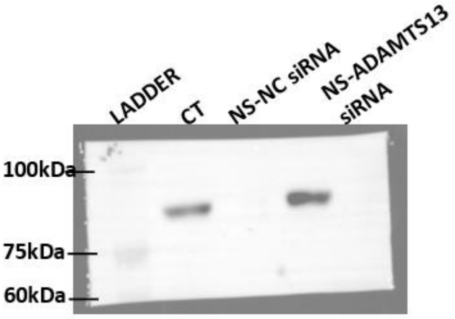

HGF-Figure 4i

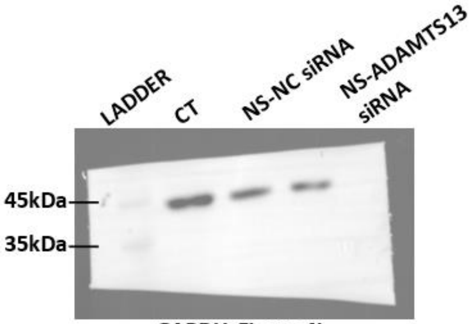

GAPDH-Figure 4i

i

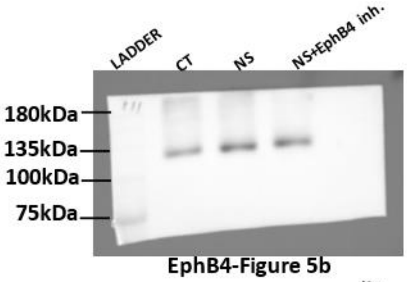

EphB4-Figure 5b

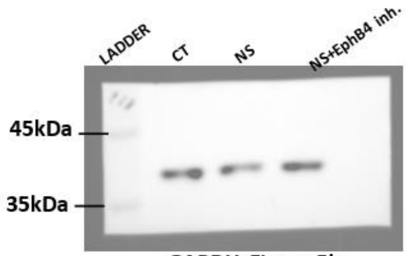

GAPDH-Figure 5b

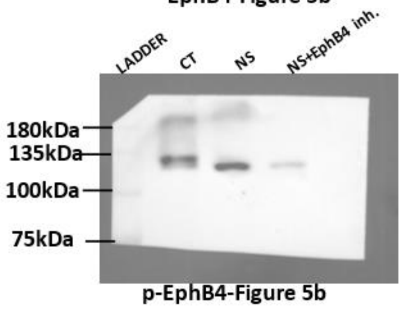

p-EphB4-Figure 5b

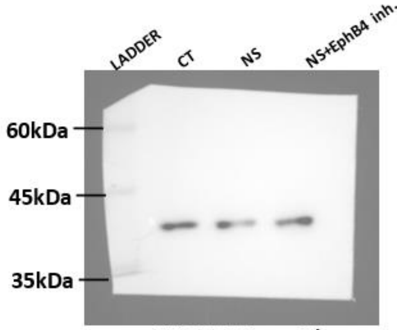

GAPDH-Figure 5b

j

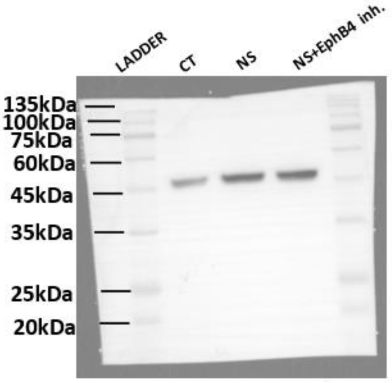

ADAMTS13-Figure 5d

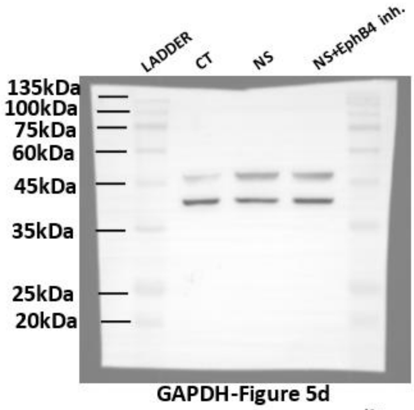

GAPDH-Figure 5d

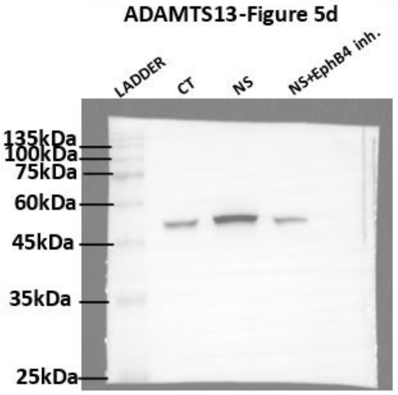

VEGF-Figure 5d

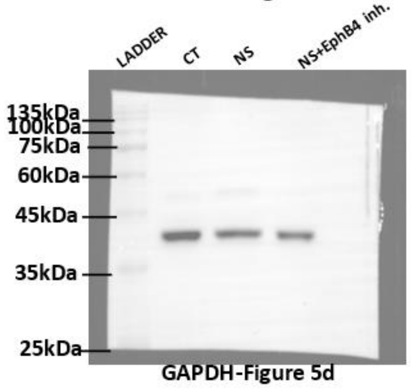

GAPDH-Figure 5d

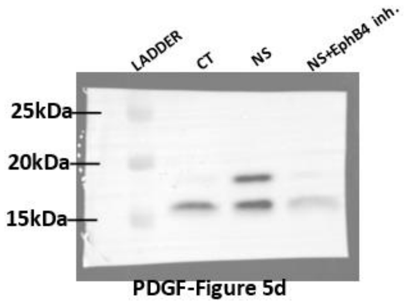

PDGF-Figure 5d

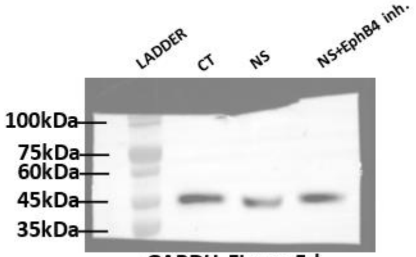

GAPDH-Figure 5d

k

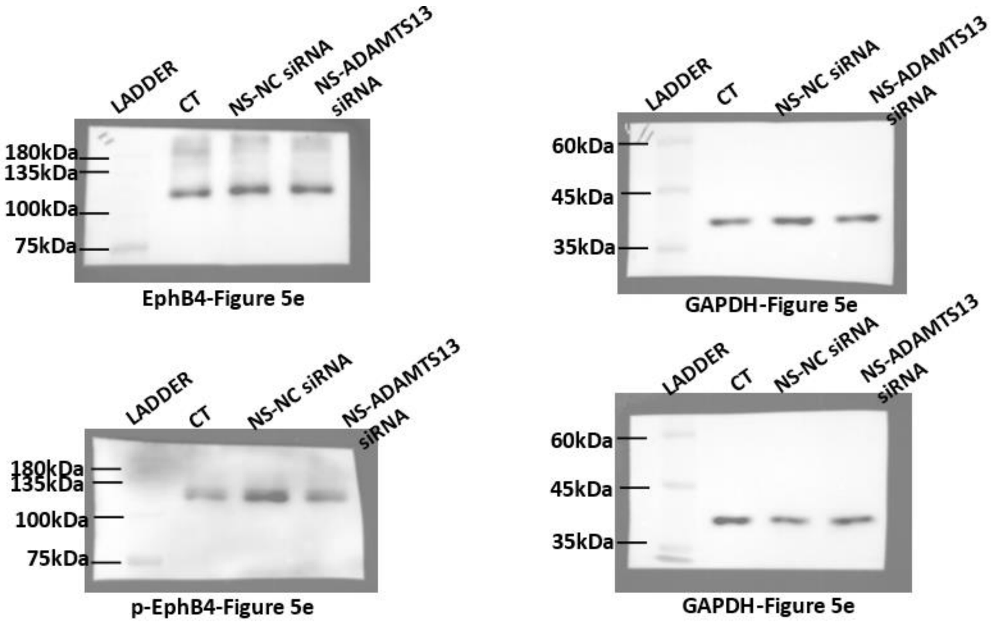
LADDER
CT
NS-NC siRNA
NS-ADAMTS13 siRNA
180kDa
135kDa
100kDa
75kDa
EphB4-Figure 5e
60kDa
45kDa
35kDa
GAPDH-Figure 5e
p-EphB4-Figure 5e
GAPDH-Figure 5e

l

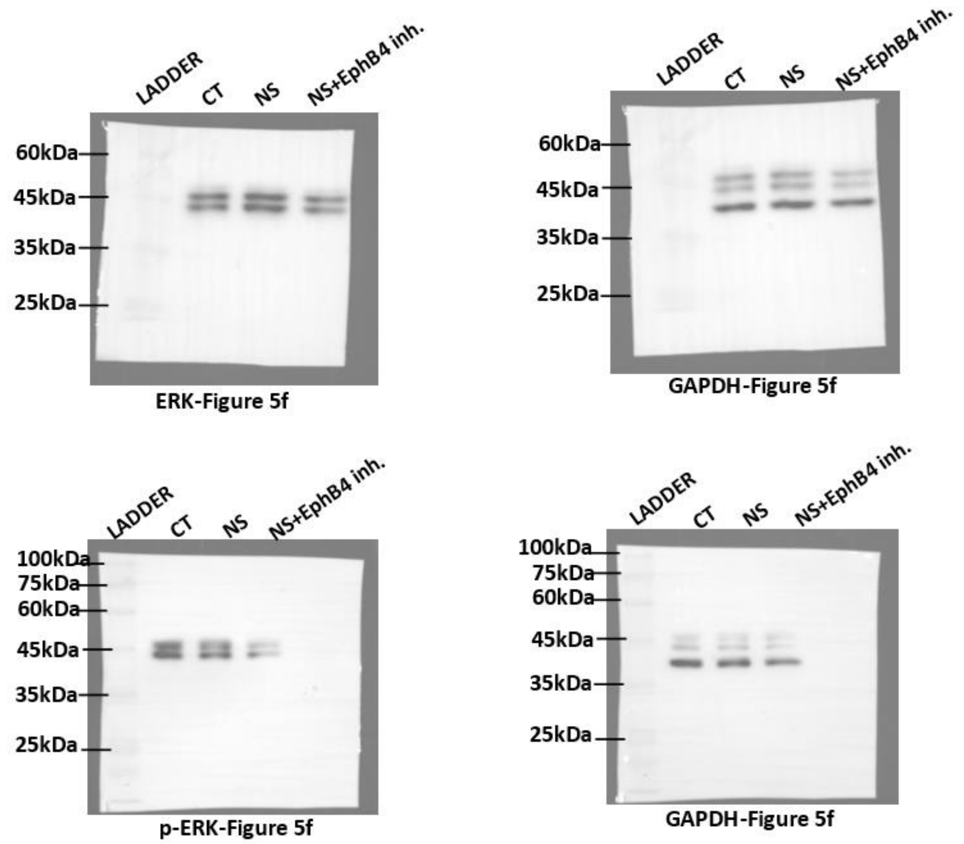
LADDER
CT
NS
NS+EphB4 inh.
100kDa
75kDa
60kDa
45kDa
35kDa
25kDa
ERK-Figure 5f
GAPDH-Figure 5f
p-ERK-Figure 5f
GAPDH-Figure 5f

m

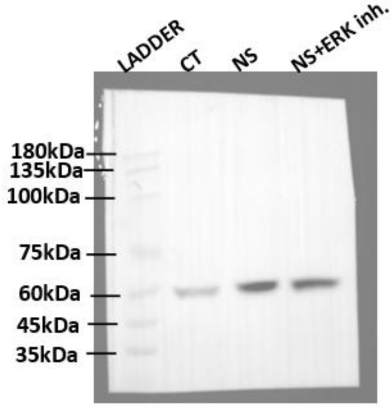

ADAMTS13-Figure 5h

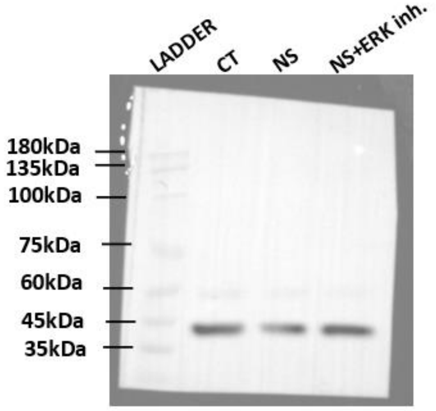

GAPDH-Figure 5h

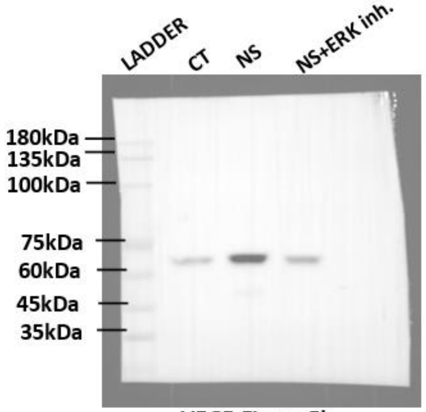

VEGF-Figure 5h

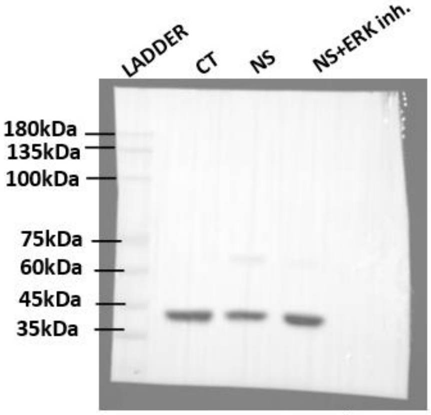

GAPDH-Figure 5h

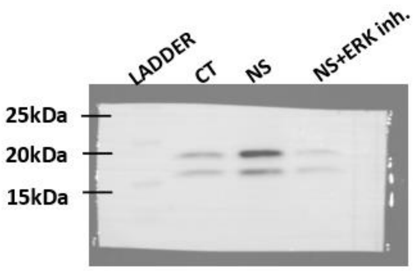

PDGF-Figure 5h

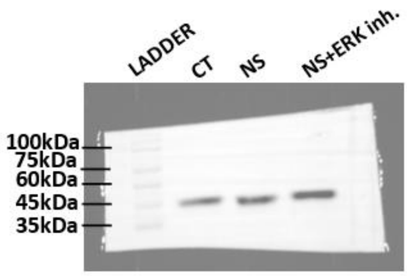

GAPDH-Figure 5h

n

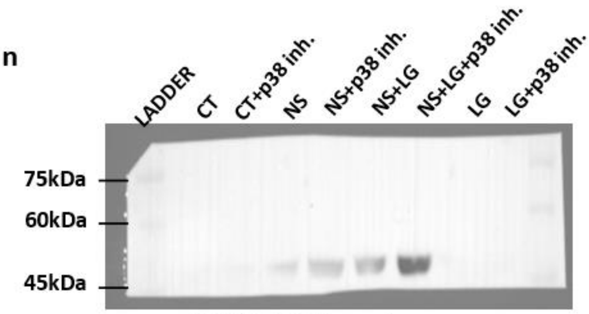

ADAMTS13-Figure 6c

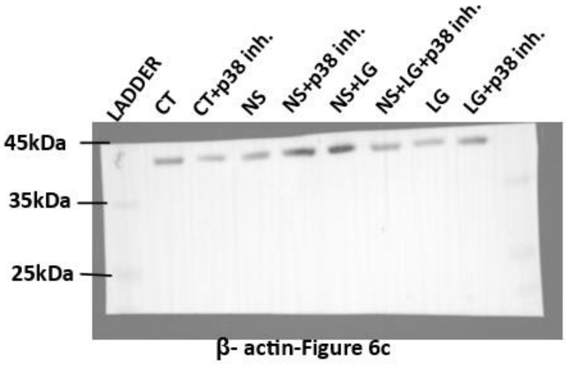

β- actin-Figure 6c

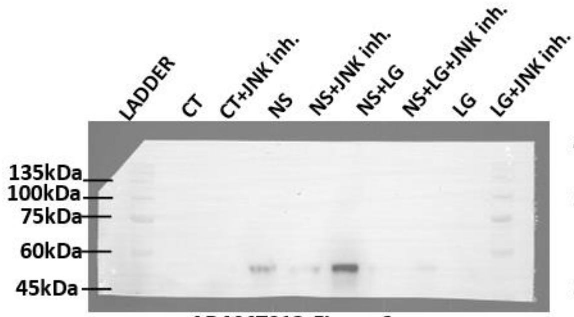

ADAMTS13-Figure 6c

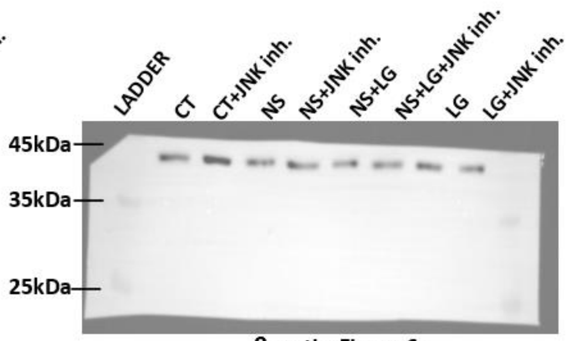

β- actin-Figure 6c

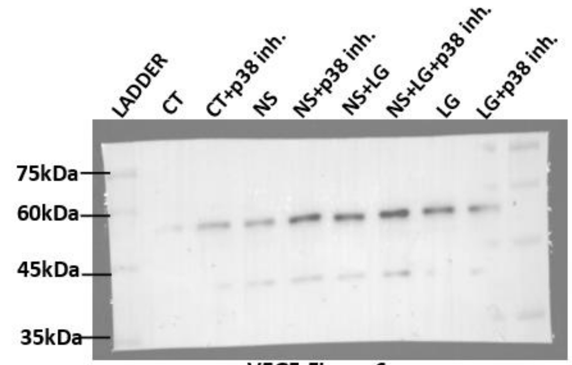

VEGF-Figure 6c

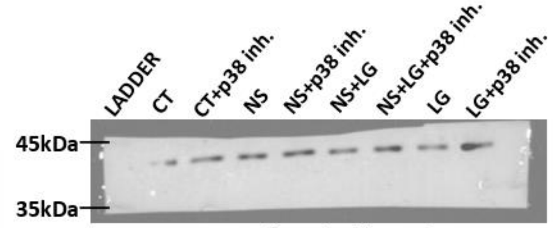

β- actin-Figure 6c

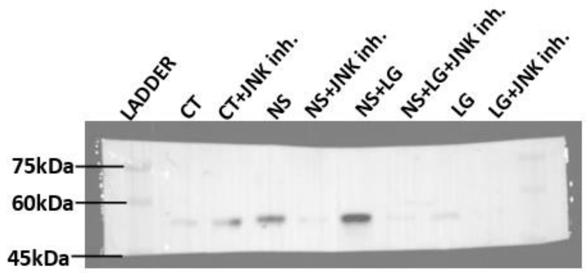

VEGF-Figure 6c

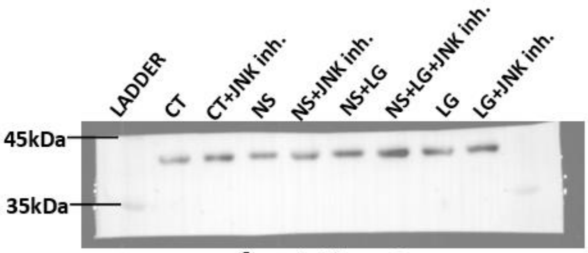

β- actin-Figure 6c

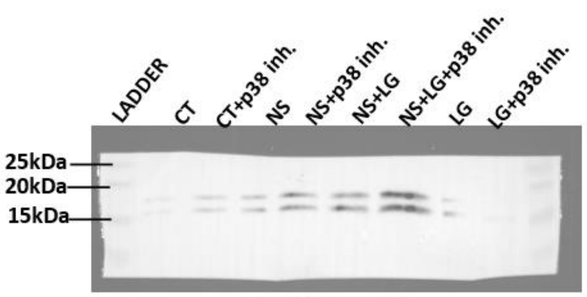

PDGF-Figure 6c

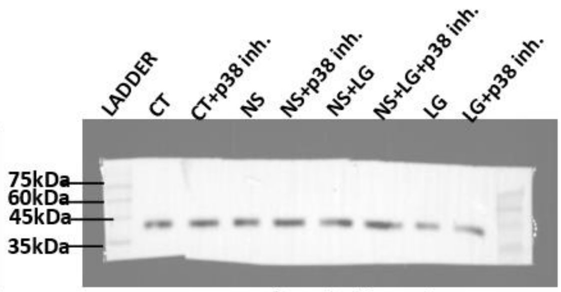

β- actin-Figure 6c

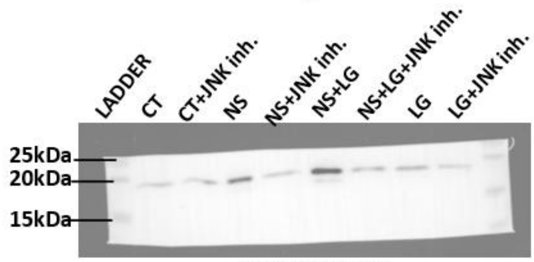

PDGF-Figure 6c

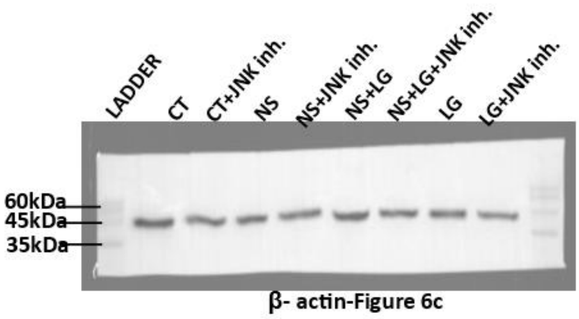

β- actin-Figure 6c

o

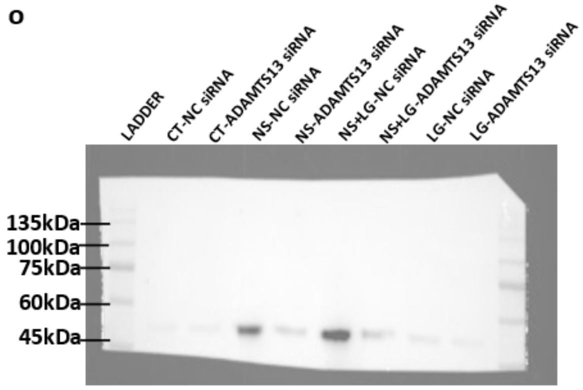

ADAMTS13-Figure 6d

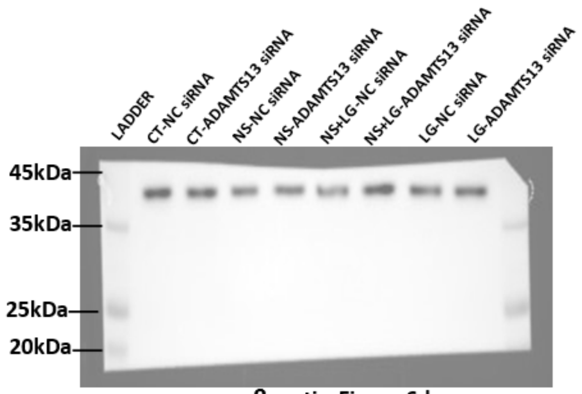

β- actin-Figure 6d

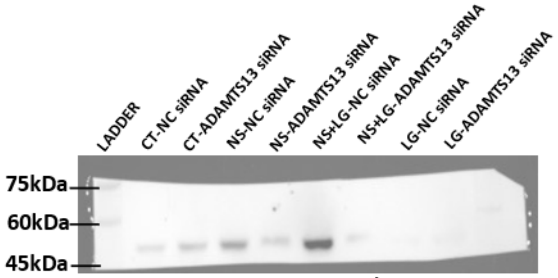

VEGF-Figure 6d

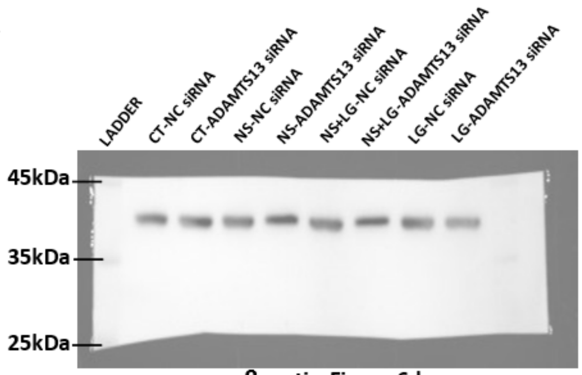

β- actin-Figure 6d

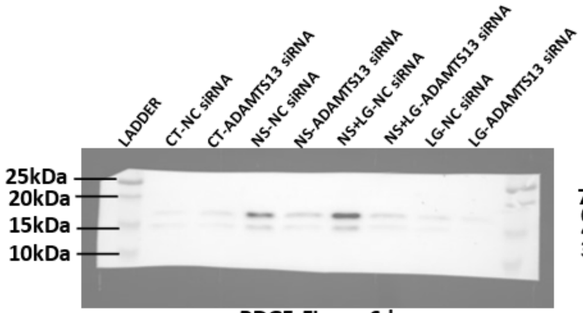

PDGF-Figure 6d

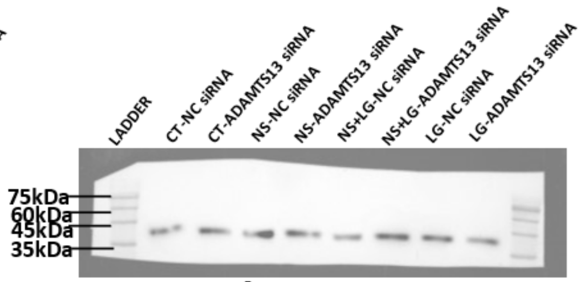

β- actin-Figure 6d
